# Simple changes of individual studies can improve the reproducibility of the biomedical scientific process as a whole

**DOI:** 10.1371/journal.pone.0202762

**Published:** 2018-09-12

**Authors:** Matthias Steinfath, Silvia Vogl, Norman Violet, Franziska Schwarz, Hans Mielke, Thomas Selhorst, Matthias Greiner, Gilbert Schönfelder

**Affiliations:** 1 German Federal Institute for Risk Assessment, Berlin, Germany; 2 Department of Endocrinology, Diabetes and Nutrition, Center for Cardiovascular Research, Charité-Universitaetsmedizin Berlin, Berlin, Germany; 3 Charité-Universitaetsmedizin Berlin, Berlin, Germany; University of Amsterdam, NETHERLANDS

## Abstract

We developed a new probabilistic model to assess the impact of recommendations rectifying the reproducibility crisis (by publishing both positive and ‘negative‘ results and increasing statistical power) on competing objectives, such as discovering causal relationships, avoiding publishing false positive results, and reducing resource consumption. In contrast to recent publications our model quantifies the impact of each single suggestion not only for an individual study but especially their relation and consequences for the overall scientific process. We can prove that higher-powered experiments can save resources in the overall research process without generating excess false positives. The better the quality of the pre-study information and its exploitation, the more likely this beneficial effect is to occur. Additionally, we quantify the adverse effects of both neglecting good practices in the design and conduct of hypotheses-based research, and the omission of the publication of ‘negative‘ findings. Our contribution is a plea for adherence to or reinforcement of the good scientific practice and publication of ‘negative‘ findings.

## Introduction

Reproducibility can be defined by a number of ways [1]. In a recent ‘Perspective‘ in PLoS Biology [1] an inclusive definition of irreproducibility was adopted that encompasses the existence and propagation of one or more errors, flaws, inadequacies, or omissions that prevent replication of results. The authors estimated that the irreproducibility of published scientific data ranges from 51% to 89%, which is supported by meta-research of recent years. Here, publication bias [2,3], methodological problems [4,5], failure of direct replication [6, 7], and the lack of power [8, 9] are consistently reported. Therefore, the result of a survey conducted by the *Nature* journal is not unexpected [10]. It seems, as the scientific community is concerned about the lack of reproducibility. Here, scientists responding to the survey assume that the reproducibility of published scientific studies is less than expected and desired. Specifically, 90% of 1,576 scientist respondents agreed there is a significant or slight reproducibility crisis. Interestingly, although published data cannot be reproduced, 31% of the respondents still do not believe that the published results could be wrong [10].

A lack of reproducibility often impairs the credibility of science and causes severe concerns of resource waste (e.g., money, time, human and animal resources), hindrance of the scientific progress and of new medical therapies, as well as the unnecessary suffering of experimental animals and wasting of a large number of animal lives, since many of the translational and preclinical studies in biomedical research are based on animal experiments. Therefore, the scientific community craves to find a solution for the reproducibility crisis.

As stated above, there are many reasons why a study cannot be reproduced. Of course, an insufficient description of a method subsequently leads to irreproducible experiments and data, which is often justified by having the methods section relegated to small paragraphs in broad spectrum journals[11]. Consequently, the methods sections often do not contain crucial information for reviewers or readers to evaluate the strength of the experimental data and to draw conclusions [11]. As such, simply reporting on experimental design and methods has to be improved to avoid reproducibility and robustness imbalances in science overall. Acknowledging this, several publishers are starting to reconsider how to report experiments. For example, *Cell Press* campaigned to empowering the methods sections [11].

More importantly, even when an experiment can be repeated, irreproducibility occurs when an alleged effect (i.e., cause-effect relationship) either does not have the indicated strength or does not exist at all. In other words, reproducibility is defined as the ability to exactly repeat an experiment and obtaining an identical result within statistical error tolerances. Hence, the reverse conclusion is leading to the very relevant question: why a result cannot be reproduced if a published experiment can be repeated exactly?

Ioannidis identified a number of reasons that contribute to the non-reproducibility of most published studies in 2005 [12]. These reasons are currently intensively discussed by the scientific community, and several practical recommendations were derived to find the way out of the ‘reproducibility crisis’ [5] for the individual study. We summarize these recommendations as follows:

- Positive results from studies indicate an effect, but ‘negative’/null results show no evidence of the expected effect. The positive predictive value (PPV) is defined as the expected number of all true positive results divided by the expected number of all positive study results. Summarizing, the PPV is a measure of the credibility of science. This quantity increases with the pre-study probability, and decreases with probability of alpha and beta errors.
  A poor selection of hypotheses (effects) to be tested leads to a low degree of pre-study probability and, thus, decreases the PPV (i.e., the number of false positive results increases). Consequently, one should carefully select the hypotheses to be tested.
- A major problem addressed in literature [5] is ‘flexible design’ (i.e., differences between randomized versus observational trials are not properly considered when a study is planned) and ‘flexible data analysis’ for experiments (i.e., alternative approaches or methods exist for analyzing the same data), which increase the tendency (bias) for positive results. It is assumed that the proportion of statistically significant positive (i.e., hypothesis confirming) results is larger than anticipated when experiments would be rigorously conducted. This problem was theoretically investigated, and is also confirmed by empirical studies and meta-research (e.g., in neurosciences, the amount of statistically significant (positive) results exceeds any rational expectation) [13]. Systematic errors in the statistical evaluation of experiments in neuroscience are one reason that could lead to a bias in favor of positive results [14,15]. Another reason is the consequence and prevalence of attrition in preclinical research through biased animal removal [4]. In fact, the distribution of published p-values hints at a bias due to ‘p–hacking’, which means the data are produced or analyzed until the p-values fall below the significance level [16]. Results are therefore biased when obtained from such flexible study designs which allow the selection of the most favorable outcomes. Prospective, rigorous, and transparent experimental design, and blinding and randomization of studies are proposed prevention measures [17]. The pre-registration of studies would also be an improvement [18–20].
- Despite their potential, the probability of successfully publishing ‘negative’/null results is lower than that for positive results [13]. For instance, ‘negative’/null results repeatedly remain in lab books, on hard disks, and drawers, or are not being published because they are rejected by scientific journals. But if ‘negative’/null results become published, these results are often reported in low impact journals or as by-products of positive outcomes and are, hence, difficult to observe. Accordingly, a large number of research findings remain unnoticed. The consideration of such results is much lower for further studies and, thus, may not influence the scientific progress.
  In summary, a lack of information about ‘negative’/null results leads to publication bias, because positive effects are overestimated (e.g., in meta-studies) and false-positive results are not disproved. To avoid these effects it is suggested to publish all ‘negative’/null results [21].
- The power of the statistical tests used to analyze experiments depends mainly on sample size, which is equal to the number of animals in a study for animal experiments, and the effect size. Lower power means that the false negative rate increases, which results in a lower probability to find the true effects. Additionally, the probability of a positive finding to be true (i.e., PPV) is also decreased, because the PPV decreases as power decreases. Therefore, a prior sample analysis is recommended to achieve the appropriate power. As such, fewer but larger studies are recommended, because research findings become more likely true [22].

Indeed, all four recommendations sound very plausible for experiments conducted in parallel. Based on this prerequisite, it can be assumed that the recommended changes improve the credibility of science measured by the PPV.

Consequently, the empirical analysis of the recommendations’ impact is needed after their implementation within the scientific community. However, this approach is likely to take years. Thus, mathematical models are a useful alternative approach for the proof of concept.

Several models have been published in recent years [23–26]. It is possible to distinguish between models, which predict the behavior of scientists under a given system of incentives, and those models, which are designed to investigate the impact of this behavior on the efficiency and quality of science.

A starting point of such consideration is that there exists a difference between the interest of the individual scientists, and the interest of the public in the quality and efficiency of science. In a simplified approach the number of publications is decisive for the career prospective of a scientist. Depending on the system of incentives, the scientists can divide their resources into many small or few large studies, and they act more or less honest in order to obtain a publication. Gall et al. [27] introduce a large set of economics and social science methods for the investigation of the effects of incentive systems.

Other models focus on the impact of a changed behavior, like the use of greater sample sizes on efficiency and quality of science.

Models using evolutionary or game-theoretic approaches belong to the group investigating the impact of incentives on the behavior and are helpful for understanding how the dynamic of the scientific cognitive progress is influenced by human conflict and cooperation within a competitive situation. In other words, these models help to understand why currently ‘bad science‘ (i.e., not all four aforementioned recommendations are appropriately considered) still exists. For instance, Smaldino and McElreath [28] demonstrated that poor methodological practices are more frequent and high false discovery rates increase as a result of the current incentive structure that rewards publication. Even replication does not stop this process. According to this approach, research groups may survive (i.e., ‘evolve‘) within the scientific community on the basis of the sheer number of publishable results, which may constitute an incentive for applying poor science. Additionally, research groups maximize their fitness—but not the scientific progress—when spending most of their effort seeking novel results and conduct small studies that have only 10%–40% statistical power. This was recently demonstrated by Higginson and Munafò [24].

Other models [23, 29, 30] serve as a formal framework for reasoning about the normative structure of science. They help to determine standards for research practice (e.g., sample sizes) and are used to optimize certain target variables (e.g., the PPV). While Bakker [30] models the causes and effects of questionable research that leads to biased effect strengths, two models with similar structure [23, 29] have investigated the impact of both: the likelihood to replicate an experiment and of publishing ‘negative’/null results on the quality of science. Whereas Nissen et al. [29] focused on a single claim at a time, McElreath and Smaldino [23] analyzed the progress of a group of scientists testing a suite of hypotheses. Concerning the impact of publishing ‘negative’/null results the studies of McElreath and Smaldino [23] on one hand and Nissen et al. [29] on the other came to contradictory results. Both models were motivated by the concern that numerous published research findings are false, as pointed out by Ioannidis [12].

De Winter & Happee [25] and van Assen et al. [26] considered the impact of ‘selective publishing’ vs. ‘publishing everything’ in a meta analytical process, where one effect is investigated until the effect strength is determined with sufficient certainty. These authors make conflicted statements on the efficiency of the publication of negative results.

Ioannidis [12] defined a model for the testing of different relationships. In his model testing of the different relationships has no influence on each other, like tests would be conducted in parallel. Ioannidis model is governed by the following parameters: pre-study odds, significance level, the statistical power, and bias. Here, the proportion of true positive and false positive results is calculated based on these assumptions [12]. However, Ioannidis does not consider the impact of a single study result on further research, i.e. whether it causes or prevents studies on new relationships. Therefore, we were looking for an extended mathematical model, which covers the consequences of each of the above mentioned recommendations (see A-D) on the scientific process as a whole. A scientific process as a whole is defined as a sequence of efforts to solve a single research problem.

In our mathematical model two relevant aspects of the scientific process are addressed:

I) Published experimental results will cause or forestall further experiments concerning the same research problem, although aiming at different potential causal relationships (in worst case: ‘bad science paves the way for more bad science’). Unpublished results, of course consume time and resources. But, they cannot have further impact on the research of other teams, because they are not perceived. II) Because of limited resources (e.g., number of laboratory animals or study participants) the desired model should explicitly consider the costs or consumption of resources, most importantly the use of experimental animals (‘bad science is a waste of laboratory animals‘).

It is important to consider especially these two aspects, as the impact of the recommendations should be measured on three objectives in an extended model:

- uncovering the true causal connection (sensitivity),
- avoiding false positive results (specificity), and
- minimizing the overall consumptions of resources.

To do so, we propose a novel mathematical model of the research and publication process, which exceeds the examination of parallel testing of hypotheses, and considers the impact of study results on consecutive research. Our extended model evaluates the impact of the aforementioned recommendations A-D on the overall scientific process (i.e., gain of knowledge), and addresses the three above mentioned objectives (a-c). These objectives are represented by three dependent variables: The scientific gain as a measure for the scientific progress (a), the number of false positives (b), and the total number of samples, which should be reduced (c).

## Model

The entire process of hypotheses testing research, encompassing consecutive studies and publications, is described in our probabilistic model. To better explain our model, we start with the simple and idealized case (initial model). Extensions of the initial model will be introduced thereafter.

An exemplary case is a single research team that explores an observed effect, such as the triggering of a signaling cascade by a certain small molecule. Based on pre-study knowledge, two scientific hypotheses will be examined: the small molecule ‘stimulates receptor A’ or ‘inhibits receptor B’. In addition, we assume that this particular research problem and its results are interesting or relevant enough for the scientific community. For instance, other researcher teams are investigating ‘compounds that stimulate receptor A’, and they might want to use ‘the small molecule’ as a positive control for their experiment.

The research team will now begin to statistically plan an experiment that covers the most likely scientific hypothesis based on their pre-study knowledge. Here, the single scientific hypothesis will be tested as an alternative against a null-hypothesis according to Neyman-Pearson [31].

Let us assume, ‘the small molecule stimulates receptor A’ is the first scientific hypothesis. To calculate the sample size needed for the experiment, the team decides for significance level **α** and statistical power (1-**β**), given the expected effect size **δ**.

After completing the experiment, a statistical test evaluates the null hypothesis.–At this point it is important to note the difference between the scientific and the statistical hypothesis. To achieve a better distinction we use the term ‘factor’ for scientific hypothesis.- The evaluation of the null hypothesis leads to its rejection (‘small molecule stimulates receptor A’) or its acceptance (‘ small molecule does not stimulate receptor A’). Furthermore note, that a rejection of the null hypothesis is not synonymous to an acceptance of the alternative scientific hypothesis (‘small molecule inhibits receptor B’).

**Fig 1:**
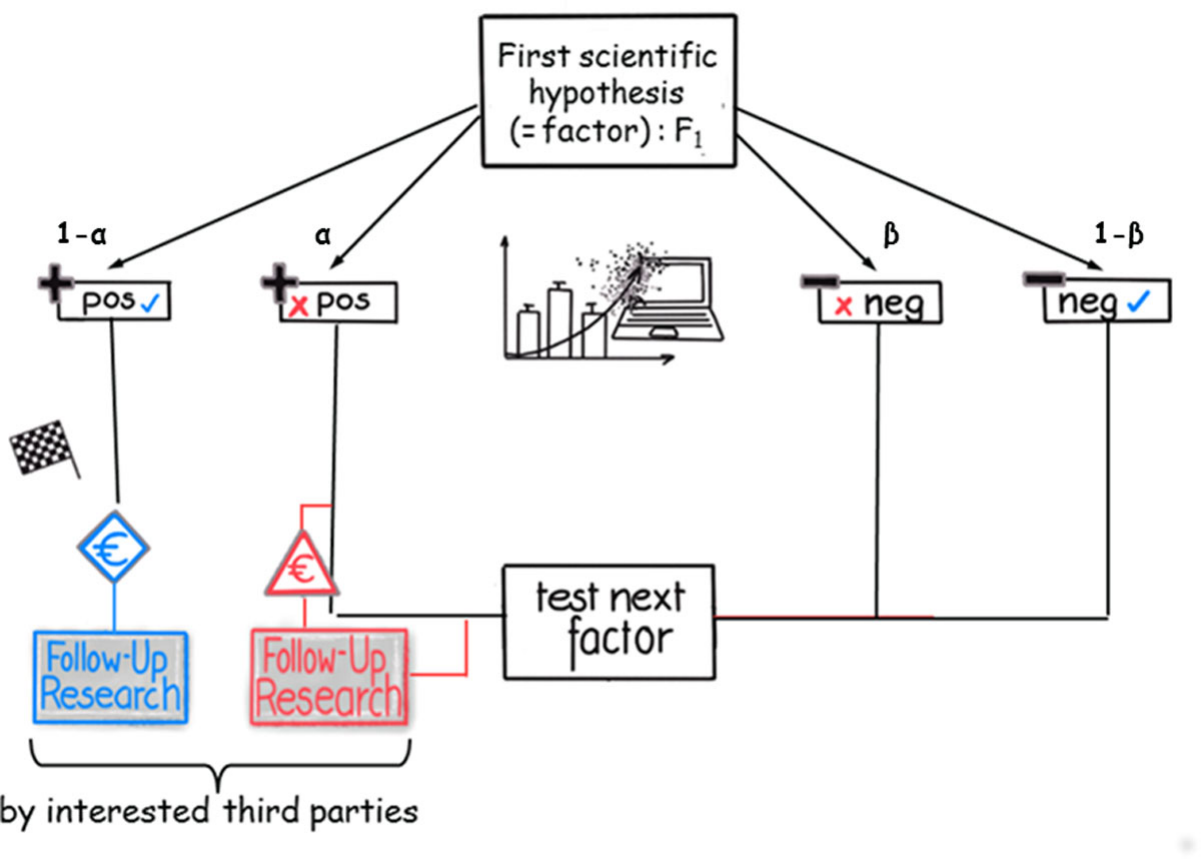
Flow diagram of the modeled research and publication process (part one). Different possible sequences of outcomes, actions, and decisions are displayed. One research team investigates the first factor, F_1_. After experiment completion, the statistical test yields positive (left side) or ‘negative’/null results (right side). These results can either be true (blue checkmark) or false (red cross). In the case of true positive results, the research problem is solved and third parties can (successfully) use the results, while false positive results can be misleading for third parties, costly, and possibly dangerously. ‘Negative’/null results lead to an exclusion of the factor from further investigations; as a consequence, factor F_2_ is tested next. In case of a false negative result, this true (causal) factor F_1_ is overlooked and, after the investigation of all remaining factors, the research process ends without a discovery.

We now imagine that the research team detects that ‘a small molecule stimulates receptor A’. The research team believes the scientific question to be solved and considers no further trials, but does not know if the result is true or false positive. As the outcome of a statistical test can either be true or false, there are four different possible results [31]: true positive, false positive, true negative, and false negative (Fig 1). The distribution of these results depends on the probability of statistical errors **α** and **β**. They lead to different overall outcomes for the research problem (answering the question of the mechanism by which the small molecule triggers the signaling cascade).

Consequently, the research team will not test the second factor, which is, their second scientific hypothesis (‘the small molecule inhibits receptor B’) and will publish the result that ‘the small molecule stimulates receptor A’. A typical scenario of science, because in general all positive results will be published.

In the long run, this scientific procedure lead to an important difference between a publication of a true positive and a false positive result: subsequent research is based on or uses the positive result, and the more it is used the more it will become apparent that the original hypothesis holds, ultimately resulting in its verification, which is a basic premise in our model. The reverse is true for the false positive result, but the cost of identifying a positive result as false might be immense and—in case of animal tests or even clinical trials—not only financially, as humans or animals could be harmed in the process. The original research team will eventually realize that their originally published positive result was a false positive and will restart and test the next hypothesis (‘the small molecule inhibits receptor B’). This procedure will possibly obtain more than one false positive within the research process.

We can therefore conclude: a false positive result is always falsified. A false positive result is always detected, because based on the published false positive result a variety of different experiments (e.g., follow-up research by other research teams) will fail. Since this assumption cannot always taken for granted, we investigated the consequences of the partial removal of these basic premises (see supporting information S2 Text).

In our exemplary case the research team will also continue to test its second scientific hypothesis (‘the small molecule inhibits receptor B’) if the statistical analysis of their first scientific hypothesis revealed a negative result: the detection of a negative result will allow the research team to test the second—possibly true—hypothesis to find the solution to its research problem. Publishing the negative result will have no impact, as there is only this single research team working on this particular scientific question. A false negative result will eventually lead to the end of the research process (all available hypotheses were tested) without discovery, because the true first hypothesis was rejected and the false second hypothesis will be also rejected (even a possible false positive result will ultimately be uncovered due to research based on the said result (falsified), as explained in our assumptions above).

**Fig 2:**
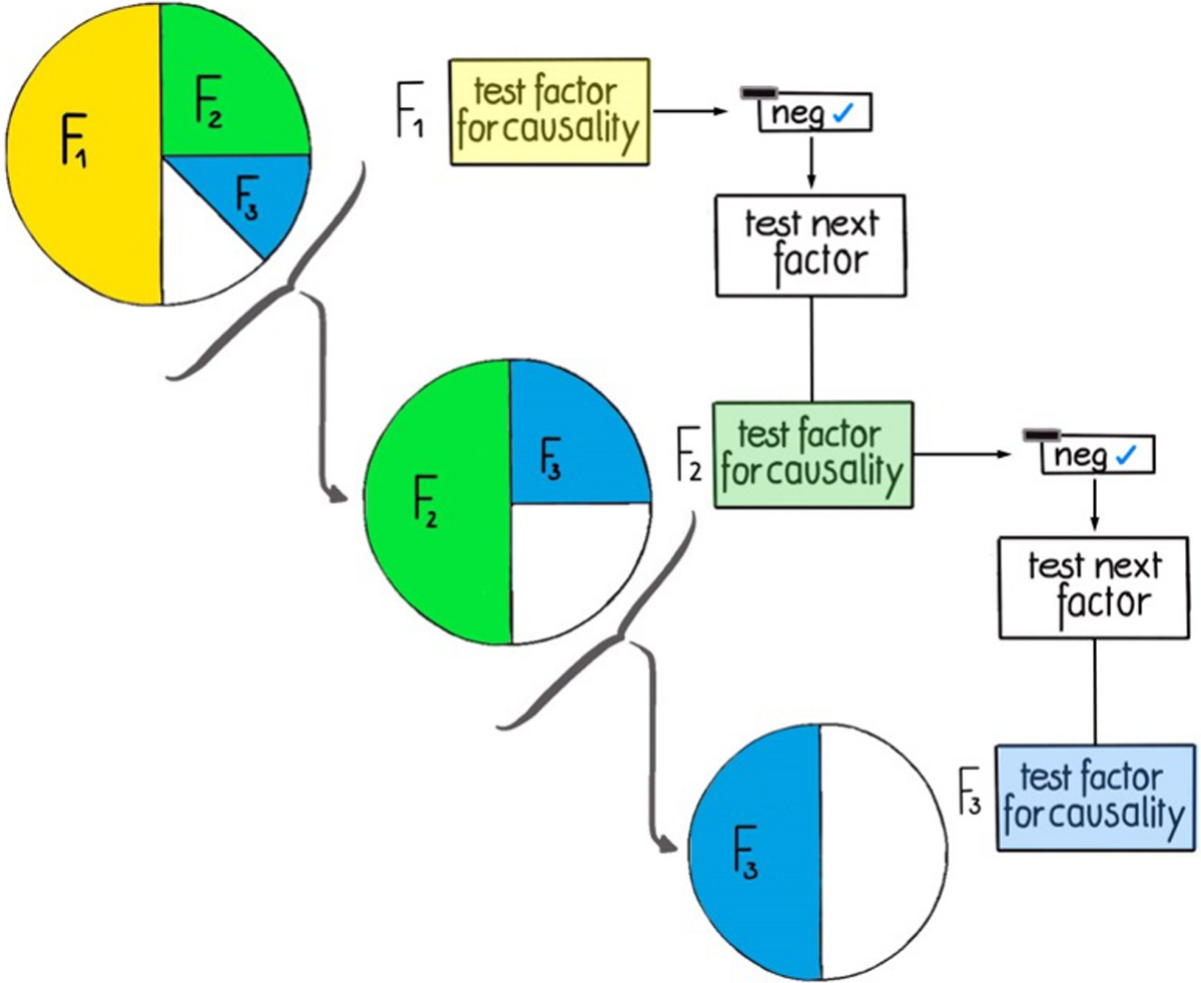
Figurative representation of how π_k_ defines pre-study probabilities. Setting **π**_**k**_ = 0.5, the pre-study probabilities **ω**_**i**_ of scientific factors F_1_, F_2_, or F_3_ to be causal are 50%, 25%, and 12.5%, respectively. If, however, F_1_ is tested and found to be not causal, the probabilities for the remaining factors will increase, the second factor F_2_ will become the most probable one, with a pre-study probability of 50%, and the pre-study probability for F_3_ will be 25%, etc.

At this point everybody would raise the question how the prioritization of the two hypotheses arose. Based on the pre-study information of course the research team selects the most likely hypothesis. This pre-study information can be comprehensive or scarce, and its availability, usage, or interpretation to create pre-study knowledge can be more or less biased. To model this, we need to include different qualities of pre-study information in a quantitative and objective manner. Consequently, we introduce pre-study probabilities **ω**_**i**_ for each factor (i.e., scientific hypothesis) **F**_**i**_, which are governed by the parameter **π**_**k**_. Fig 2 depicts an example with three factors.

**Fig 3:**
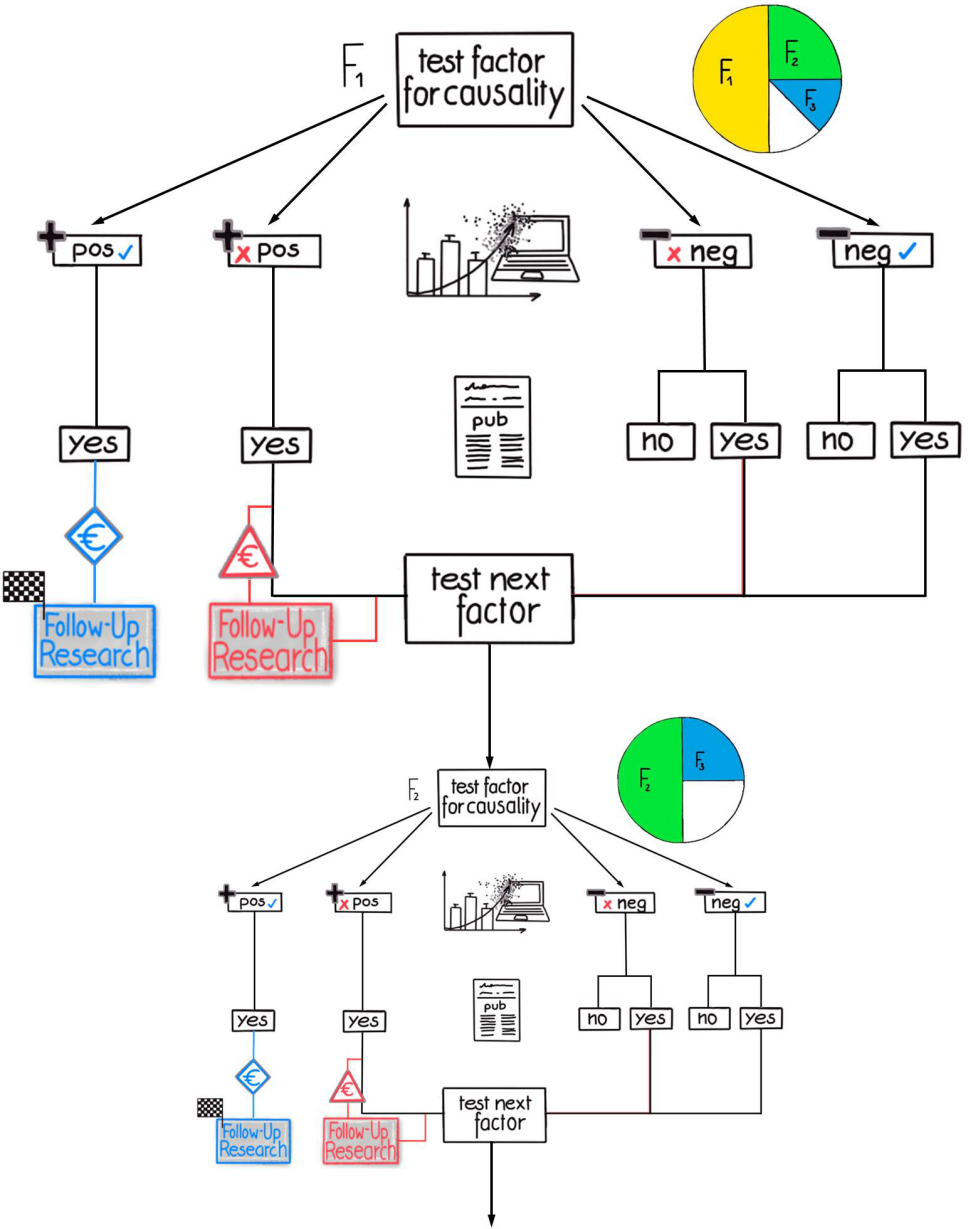
Flow diagram of the modeled research and publication process (part two). The different possible sequences of outcomes, actions, and decisions are displayed when several research teams are involved and ‘negative’/null results are published with probability **P**_**pub**_ (see Fig 1): published ‘negative’/null results have an influence on ‘competing‘ research teams so that those factors do not have to be tested more than once.

The higher **π**_**k**_ the higher the probability that the first factor in the ranking is the ‘right‘ (i.e., causal) factor. In other words, according to the formula given in the supporting information (S1 Text, equation 1): if **π**_**k**_ = 0.5, the pre-study probability of the first factor (**ω**_**1**_) to be causal is 50%, if **π**_**k**_ = 0.9, then **ω**_**1**_ = 90%. To simplify the modeling procedure, **π**_**k**_ is defined as to maintain the pre-study probability ratio constant between two consecutive ranks. This is depicted in Fig 3 (for more details, see the supporting information (S1 Text)). Therefore, **π**_**k**_ can be interpreted as the quality (i.e., predictive power) of the pre-study information available for the research problem under investigation.

In reality, even if there is high quality pre-study information, circumstances such as current research trends, financial considerations, availability of publications, and also the ‘human factor’, lead to situations in which all this theoretical available information is not fully exploited, leading to incomplete pre-study knowledge. Obviously, this can result in a ‘non-optimal‘ prioritization of research activities. Non-optimal prioritization can be understood as any sequence of research activities, which does not lead to the yielding solution to the problem. Therefore, our model covers the possibility that factors might be tested in a ‘non-optimal‘ sequence (i.e., not in the order defined by their pre-study probabilities). Applied to our exemplary case, i.e. the research team first investigates the scientific hypothesis where ‘the small molecule inhibits receptor B’ (second hypothesis, F2), because receptor B seems to be more interesting.

The research team statistically plans the first experiment and calculates the needed sample size by setting the significance level **α**, and the statistical power (1-**β**). Additionally, the resulting sample size also depends on the effect size **δ.** This effect size should the one deemed relevant for question under investigation and is often estimated based on pre-study knowledge. Overestimation of the effect size would lead to a decrease of statistical power, because of previous underpowered studies. For simplicity, we assume that the effect size is estimated correctly. For the same reason, the sample size calculation is used for all factors for one research problem, and all factors considered for a research problem have either zero-effect strength (non-causal factors) or the same finite effect strength (causal factors).

Considering game-theoretical approaches, we incorporated in our model also competing research teams, which are also interested in the same research question. Simplified, we have two competing research teams (team 1 and team 2). Both teams use the same pre-study information and come to the same conclusion to test exactly the same hypotheses. As they compete, both teams only recognize all published results, but do not directly exchange information. However, team 2 may be not as fast as team 1 in preparing the experiment, because team 2 begins later for example. If team 1 publishes a positive result for receptor A in the meantime, team 2 will not start to repeat the same experiment. In contrast, if team 1 tests receptor A negatively, publishes the result not with certainty but with the probability **P**_**pub**_. Team 2 will also investigate receptor A in the case the result is not published by team 1. In this case resources will be wasted. The same procedure is repeated with each of the subsequent hypotheses tests by team 1. At this point it is important to mention, that the procedure for more than two teams can be defined analogously. Therefore, the number of teams, **n**_**t**_, involved in solving a research problem is another important parameter for the calculation of the total amount of used resources (and time).

Again, the parameter **P**_**pub**_ reflects the probability of the result from the hypothesis testing to be recognized by the scientific community. **P**_**pub**_ can approach 0 under a variety of circumstances, such as a result not being published. Or a result might not be recognized, because it is only mentioned in a subordinated clause and as a by-product of positive outcomes difficult to observe. In contrast, **P**_**pub**_ can approach 1, when published ‘negative/null’ results are totally noticed by scientific community.

The entire course of action is depicted in Fig 3.

To evaluate the robustness of our model against deviations from its basic assumption, we introduced further extensions to the model. These are explained in the following paragraphs (for details check supporting information (S2 Text)):

While the model basically postulates that the rules of good scientific practice (GSP) are followed, it also includes the possibility to deviate from GSP and thereby increase the possibility of (false) positive results. To model these deviations we introduce parameter **u**, which is defined in accordance to Ioannides [12]: the ‘*proportion of probed analyses that would not have been “research findings*,*” but nevertheless end up presented and reported as such*, *because of bias*.*’* Furthermore, we describe and apply another approach in the supporting information (S2 Text), which resembles the questionable research practices described by Bakker et al. [30]. As previously mentioned, we finally include another form of bias in our model: we postulated that the hypotheses are not tested in the order of declining pre-study probabilities, but in a completely different order (e.g., due to hypes or available technology).

Additionally, we considered further scenarios (S2 Text): In parallel to the investigation of receptor A by the team 1, team 2 may deliberately decide to investigate receptor B even if it is aware that receptor B is less likely to be causal. Therefore **n**_**p**_ was included as an additional parameter. **n**_**p**_ stands for n different teams testing the number of factors in parallel.

In our model, we vary **β** and **P**_**pub**_ to quantify their impacts on the overall number of true positives (which designates the scientific gain, **g**), the overall number of false positive results **fp**, and the amount of laboratory resources needed for the entire research process. In the following, we use the total number of samples needed (**n**_**total**_) as a measurement for those resources (S1 Text, equations 15, 17, and 18).

Since this study is about statistics and probabilities, actually their expectation values are considered and denoted as **E{g}**, **E{fp}**, and **E{n**_**total**_**}**. Expectation values are theoretical mean values. For example the scientific gain **E{g}** is a real number between 0 and 1, in scenarios with only one true hypothesis.

## Results

In this paper, we focus on the effects of statistical power (1-**β**) and probability to publish ‘negative’/null results (**P**_**pub**_) on the scientific gain (**g**), number of false positives (**fp**), and total number of samples (**n**_**total**_) required throughout the entire research process. Additionally, the consequences of scientifically biased experimental approaches are investigated by introducing and varying bias parameter **u** as explained in the last paragraph [12]. We also examined scenarios in which the potential factors are tested in a ‘non-optimal ‘ or biased order. As stated in the previous section, all effects on **g**, **fp**, and **n**_**total**_ are determined by calculating their corresponding expected values (**E{g}**, **E{fp}**, and **E{n**_**total**_**}**).

### Systematic analysis of the influence of statistical power

Here, we are especially interested in the question of whether the competition between our three objectives a-c (see introduction) can be partially overcome. First, let us examine significance level **α**. In the initial model, **α** has an impact only on both the number of false positives **E{fp}** and the selection of sample size **n**_**a**_, although in opposite directions: while **E{fp}** increases with **α**, the sample size **n**_**a**_ decreases with **α**. Consequently, either **E{fp}** or **E{n**_**total**_**}** can be minimized when only **α** is varied. The solution is to choose a value of **α** to limit the number of **fp**, hold it constant, and then vary statistical power (1-**β**) to address only this question properly. If not mentioned otherwise, a value of **α** = 0.05 is used in this contribution.

The influence of statistical power (1-**β**) on the reliability of experimental results in a single experiment is well known: The higher the power the lower the possibility to miss a causal factor. We can show that this aspect holds true even if different values of **u** and **P**_**pub**_ are considered. Hence, the statement applies for entire research processes: with an increasing power (1-**β**) the scientific gain (**E{g}**) increases monotonically. This relationship can be proven with the derivative of the corresponding model equation (see supporting information S1 Text).

It is also established that the false discovery rate, that is, the number of published false positive results divided by the number of all positive results, decreases with (1-**β**)[8]. Additionally, using the equations deduced from our model (see supporting information S1 Text), we can show that even the absolute number of published false positive results (**E{fp}**) decreases during the research process. This means, that less false positive results will be obtained with an increase in power (1-**β**). Therefore, no conflict between optimizing **E{g}** and **E{fp}** exists, and, consequently, increasing the statistical power has only positive effects in this framework.

**Fig 4:**
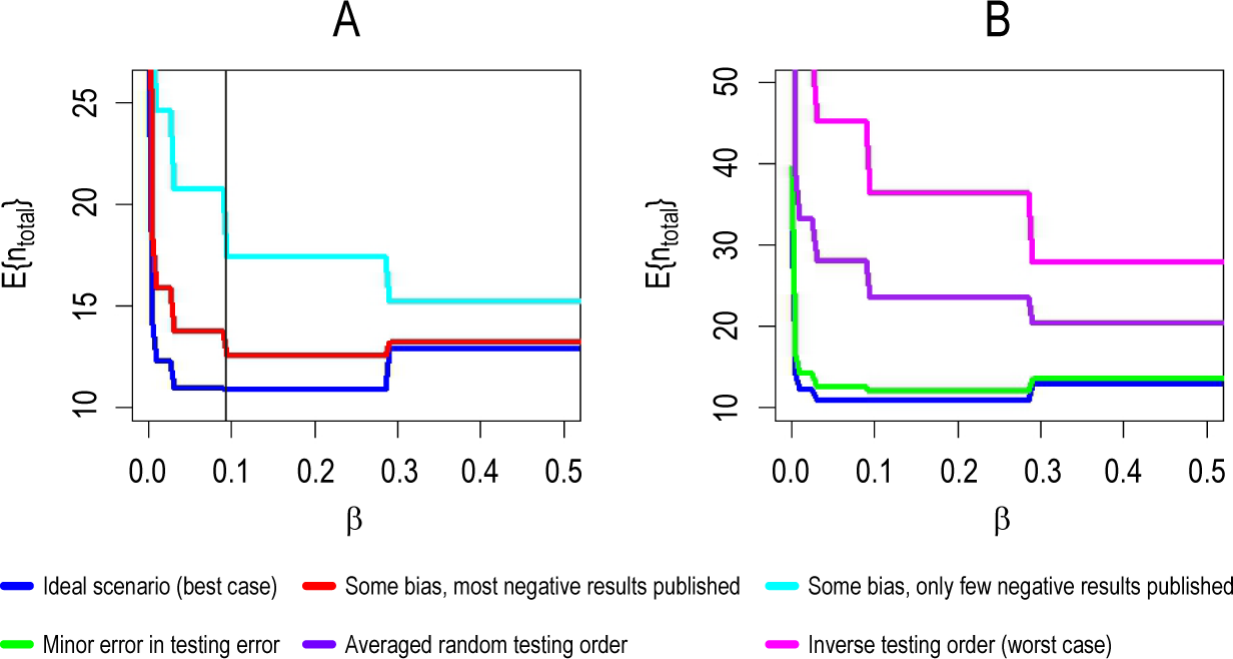
Total number of samples as function of β (= 1power). While **E{n**_**total**_**}** could be depicted as a continuous function of **β**, we present **E{n**_**total**_**}** as a step function. This is due to samples being often indivisible units, such as biological replicates or animals, so that the calculated sample size has to be rounded up to the next integer. Panel A compares different combinations of values for **P**_**pub**_ and bias **u**, whereas Panel B illustrates the consequences of changing the order in which the factors under investigation are examined. In both panels, the ideal scenario is represented in dark blue (**P**_**pub**_ = 1, **u** = 0, ‘correct order‘). **E{n**_**total**_**}** is minimized at **β**_**min**_ = 0.095 (marked by a black vertical line). **Panel A**: the red line represents deviation from ideal scenario (**P**_**pub**_ = 0.5, **u** = 0.2: only 50% of ‘negative’/null results are published and 20% excess false positive results were produced by bias). The cyan line represents even more unfavorable conditions (**P**_**pub**_ = 0.1, **u** = 0.2) without useful minimum. **Panel B**: the green line represents a minor error in the testing order, that is, testing the second most probable factor before the first most probable factor. The dark purple line shows the average random order (i.e., all possible testing orders were examined); and the magenta line represents the ‘worst case’, that is, the inverse testing order. Fixed parameters: **π**_**k**_ = 0.5, **n**_**F**_ = 10, **n**_**t**_ = 10, **α** = 0.05, **δ** = 2; **n**_**t**_ and **n**_**F**_ define the number of teams and the number of investigated factors in the research process, respectively. The sample size was calculated for one-sample one-sided t-tests.

Once again, let us look at a single experiment where scientists calculate the matching sample size for a targeted statistical power. Here, an increase of statistical power always leads to a higher sample size. What happens if we take the entire research process into account? Applied to our model, the answer is remarkable: **E{n**_**total**_**}** is not necessarily a monotonic function of **β**. The consequence of this statement is, that even if the sample size of a certain single experiment increases with the statistical power, the expected number of studies, and, therefore, the total number of samples **E{n**_**total**_**}** needed to solve the research problem can decrease. Hence, an intriguing question arises: are there useful local or global minima of **E{n**_**total**_**(β)}** so that there is a value **β**_**min**_ that minimizes the total number of samples needed to find the true causal factor, without generating an excess of false positives? To answer this question we have to examine all possible scenarios for **E{n**_**total**_**(β)}**. Some conclusive examples can be found in Fig 4. The left-hand diagram (panel A) compares different combinations of values for **P**_**pub**_ and bias **u**. The right-hand diagram (panel B) illustrates the consequences of that type of bias, where the order in which the factors under investigation are examined is changed. In both panels, the lower line (dark blue) represents the scenario, in which all results are unbiased and published (**P**_**pub**_ = 1, **u** = 0) and are obtained in the correct order. While **E{n**_**total**_} could be depicted as a continuous function of β, we present **E{n**_**total**_**}** as a step function. This is due to samples often being indivisible units, such as biological replicates or animals, so that the calculated sample size has to be rounded up to the next integer.

In panel A, the non-monotonic curve progression is very pronounced in the ideal scenario. Therefore, a minimum exists (depicted by the vertical line near **β** = 0.1). Remarkably, its location on the x-axis is independent from the actual parameter combination, although the absolute number of samples at **β**_**min**_ differs. As such, any deviation from this ideal scenario leads to an increase of **E{n**_**total**_**}** and, eventually, also to the disappearance of the (useful) minimum.

If pre-study knowledge is not sufficiently exploited, the useful minimum also disappears, which is depicted in panel B. One instance represents a minor error in the experimental order with small differences from the ideal scenario, whereas another two instances does not show an existing minimum at all; that is an averaged random order and the worst-case inverse order, respectively. Note the different y-axis scale in the two panels. More details are given in the figure caption.

Obviously, any low sample size that minimizes **E{n**_**total**_**(β)}**—being a function of **n**_**a**_**(β**_**min**_**)**—must be rejected, since choosing such a sample size would worsen the outcome of the research process in all three respects: **E{fp}**, **E{g}**, and **E{n**_**total**_**}**. In other words, a lower power than (1-**β**_**min**_)—in Fig 4, to the right of **β**_**min**_—should be avoided.

Higher sample sizes could still be chosen, since this would decrease **E{fp}** and increase **E{g}**. However, increasing higher sample sizes leads only to ever smaller gains. Therefore, it is advisable to set a sensible upper limit for the sample size **n**_**a**_. For example, the maximum efficiency—defined by **E{g}/E{n**_**total**_**}**—can be used to find such limit. Global or local minima of **E{n**_**total**_**(β)}** can then be defined as useful if they are close to the global efficiency maximum.

In conclusion, such useful values for **β** that minimize the total number of samples exist, when the entire research process is considered. Small deviations from the ideal scenario do not change **β**_**min**_. However, these useful minima can be lost, if more unfavorable conditions are assumed. This may, for example, be the case if only a small percentage of ‘negative’/null results are published or bias leads to some excess of false positive results. Insufficient exploitation of pre-study information can also result in a loss of useful minima.

With regard to the existence of these minima (**β**_**min**_), our model demonstrates further important facts. The value of **β**_**min**_ strongly depends on the quality of pre-study information, **π**_**k**,_ and the number of considered factors, **n**_**F**_. With higher values of **π**_**k**_ and **n**_**F**_, the value of (1-**β**_**min**_) also increases. In other words, the use of a higher statistical power in an experiment is especially beneficial if high quality pre-study information is available (and used) and/or more factors are considered. The proofs of these propositions are given in the supporting information (S1 Text).

The propositions render it plausible that there is a great area in the parameter space of **u**, **P**_**pub**,_ **δ**, and **n**_**p**_ (the number of teams working in parallel) for which useful minima (**β**_**min**_) exist. To investigate this question more closely, we varied these parameters and simulated the outcome, which confirmed the assumption. Details are given in the supporting information (S2 Text). In general, the area of existing useful minima **β**_**min**_ in the parameter space reduces if the research community deviates from GSP, but there is still a large such area if the deviations are small.

### Consequence of lowering α from 0.05 to 0.005

A recent publication proposes to change the default P-value threshold for statistical significance from 0.05 to 0.005 in order to improve reproducibility for claims of new discoveries[32]. Therefore, we modelled our calculations with **α** = 0.005. In brief, while the number of false positives will be reduced drastically, the total resource consumption will rise. A reduction of the statistical power is not recommended, because **β**_**min**_ will be lower in most of the scenarios investigated if **α** is decreased. The detailed results are presented in the supporting information (S2 Text).

### Difference to the Ioannidis model

**Fig 5:**
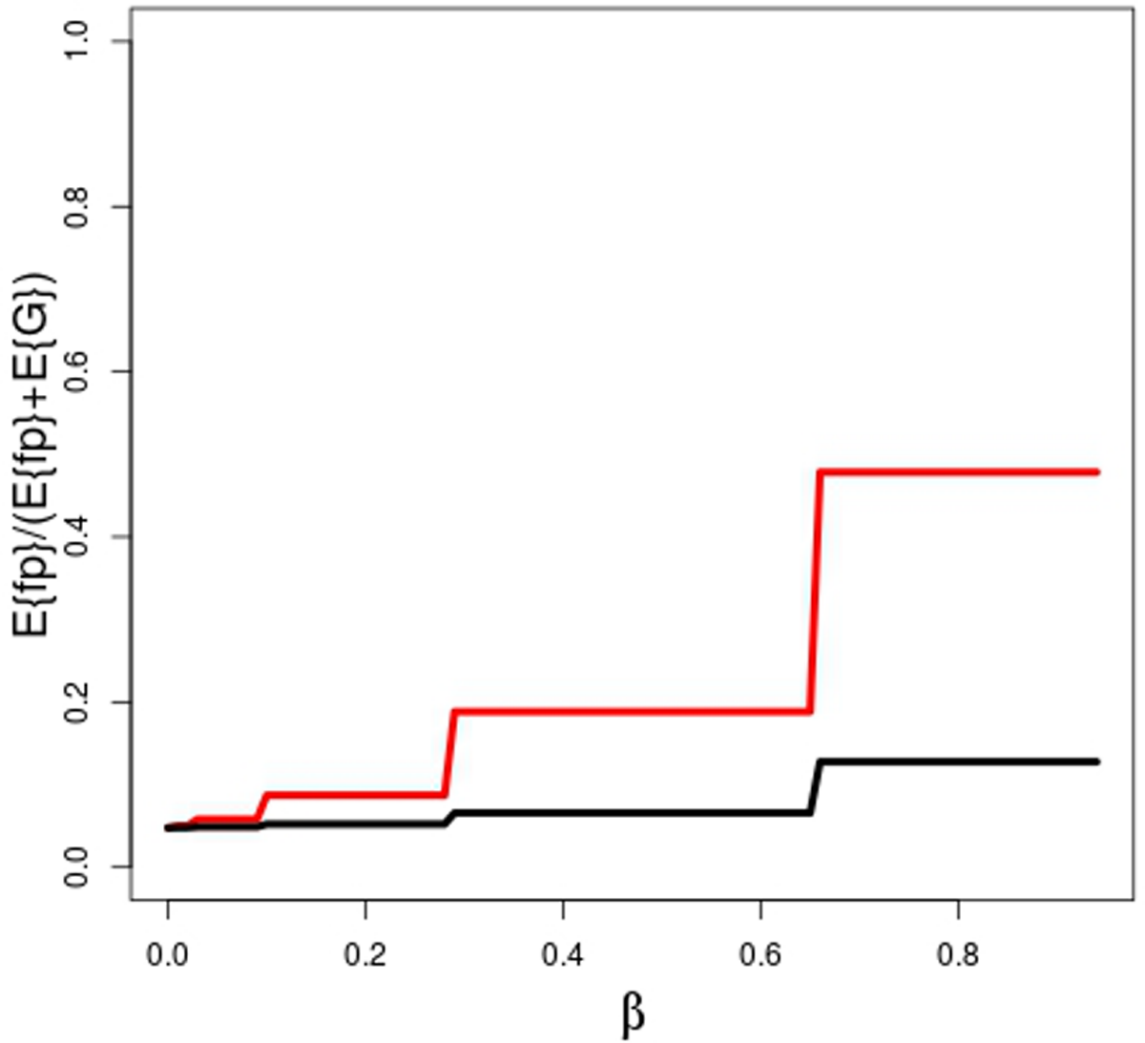
False discovery rate as function of β (= 1-power). Red line: calculation according to our model, black line: calculation according to the model to Ioannidis[12]). No publication bias (**P**_**pub**_ = 1), **π**_**k**_ **=** 0.5, **R** = **π**_**k**_/(1-**π**_**k**_) **= 1, α** = 0.05, **δ** = 2.

In the model of Ioannidis the false positive rate (**(α+u(1-α))/(R+β+βR+u–uα+uβ);** Table 2 [12]**)** depends on the statistical power. In contrast to his model our false positive rate is represented by **E{fp}**/ **(E{g}+ E{fp}) (S1 Text).** Both false positive rates are calculated for the same set of **β**. To achieve comparability we assumed all negative results are published and no bias exists (**P**_**pub**_ = 1, **u** = 0). Our analysis revealed even stronger detrimental effect of insufficient power (Fig 5).

### Systematic analysis of the influence of the probability to publish ‘negative’/null results

Interestingly, the probability to publish ‘negative’/null results (**P**_**pub**_) influences the expected number of false positive results **E{fp}** and does so in a complex manner. **E{fp}** depends on a) the number of non-causal factors that can be tested before the research process stops as explained in Fig 3 and b) on the probability for a false positive result per tested non-causal factor. **P**_**pub**_ affects both quantities differently.

If ‘negative’/null results are published–which is more likely with high **P**_**pub**_–the expected number of total experiments decreases, because non-causal factor experiments does not have to be repeated by other teams. This in turn decreases the chance of producing false positive results.

**Fig 6:**
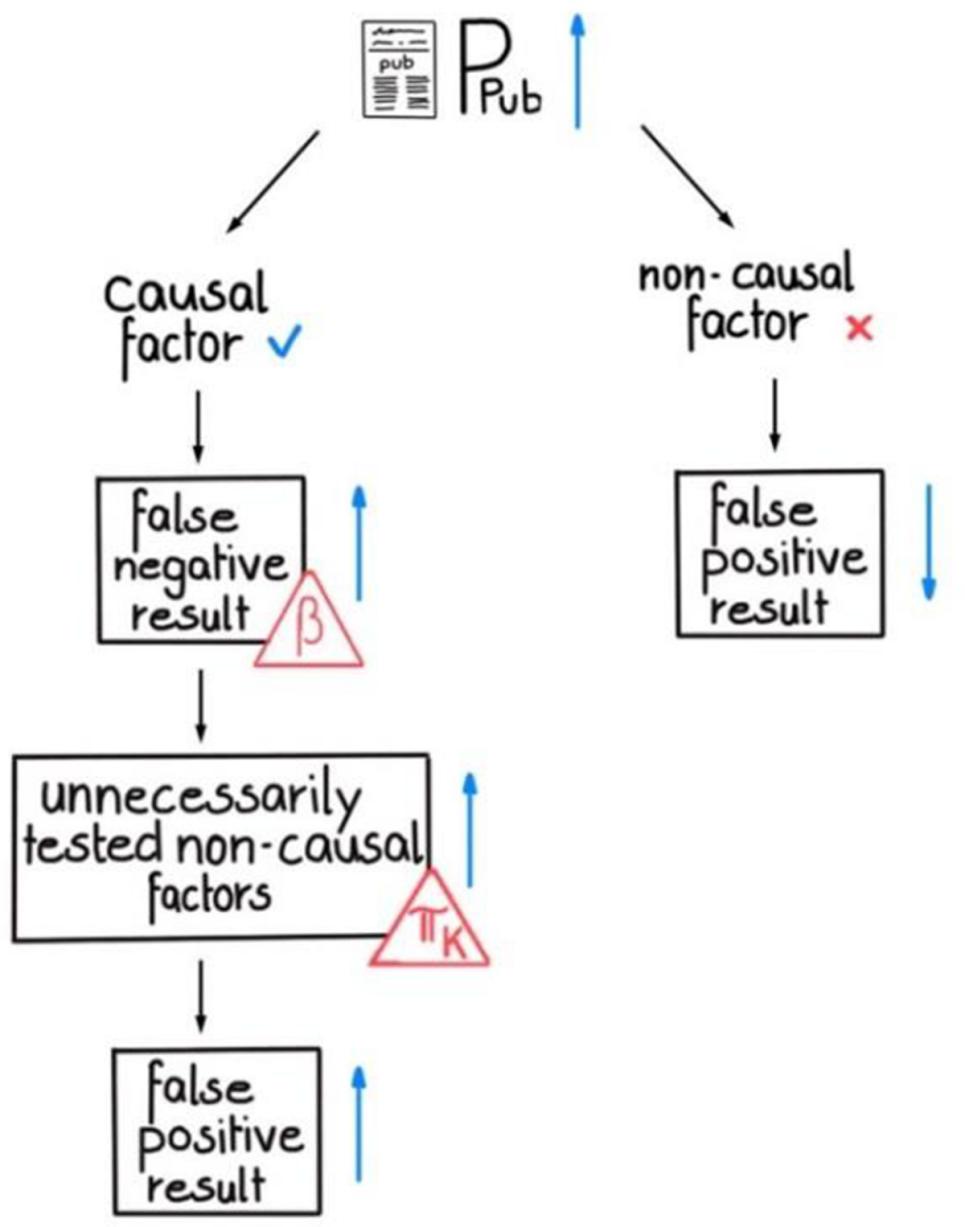
The complex influence of P_pub_ on the number of false positive results. For non-causal factors, an increase of **P**_**pub**_ leads directly to a smaller number of false positive results, because those factors are tested less often. For causal factors, an increase of **P**_**pub**_ results in a higher number of false ‘negative’/null results, which strongly depends on statistical power. However, false ‘negative’/null results lead to unnecessarily tested non-causal factors, and the higher **π**_**k**_ is, the higher the probability that the causal factor is tested in the beginning of the research process. A false negative result would therefore have a severe impact on the number of unnecessarily tested non-causal factors. This, in turn, increases the number of false positive results.

Publishing false negative results will be more likely with higher **P**_**pub**_. As a result, the number of experiments and the false positive rate increases. This outcome aggravates when the causal factor is tested early in the research process, since more non-causal factors are left to be tested. Hence, the probability for this chance depends on **π**_**k**_ (Fig 6, left). In turn, every unnecessary experiment testing a non-causal factor raises the rate of false positive results again.

This outcome aggravates when the causal factor is tested early in the research process, since more non-causal factors are left to be tested. Hence, the probability for this chance depends on **π**_**k**_ (Fig 6).

Taken together, this means **P**_**pub**_ has contrasting effects with respect to **E{fp}** on causal and non-causal factors. While a higher **P**_**pub**_ leads to a lower false positive rate, it also leads to a larger number of tested non-causal factors. Therefore, the overall effect of **P**_**pub**_ on **E{fp}** depends on which of these contrasting effects is dominant.

**Fig 7:**
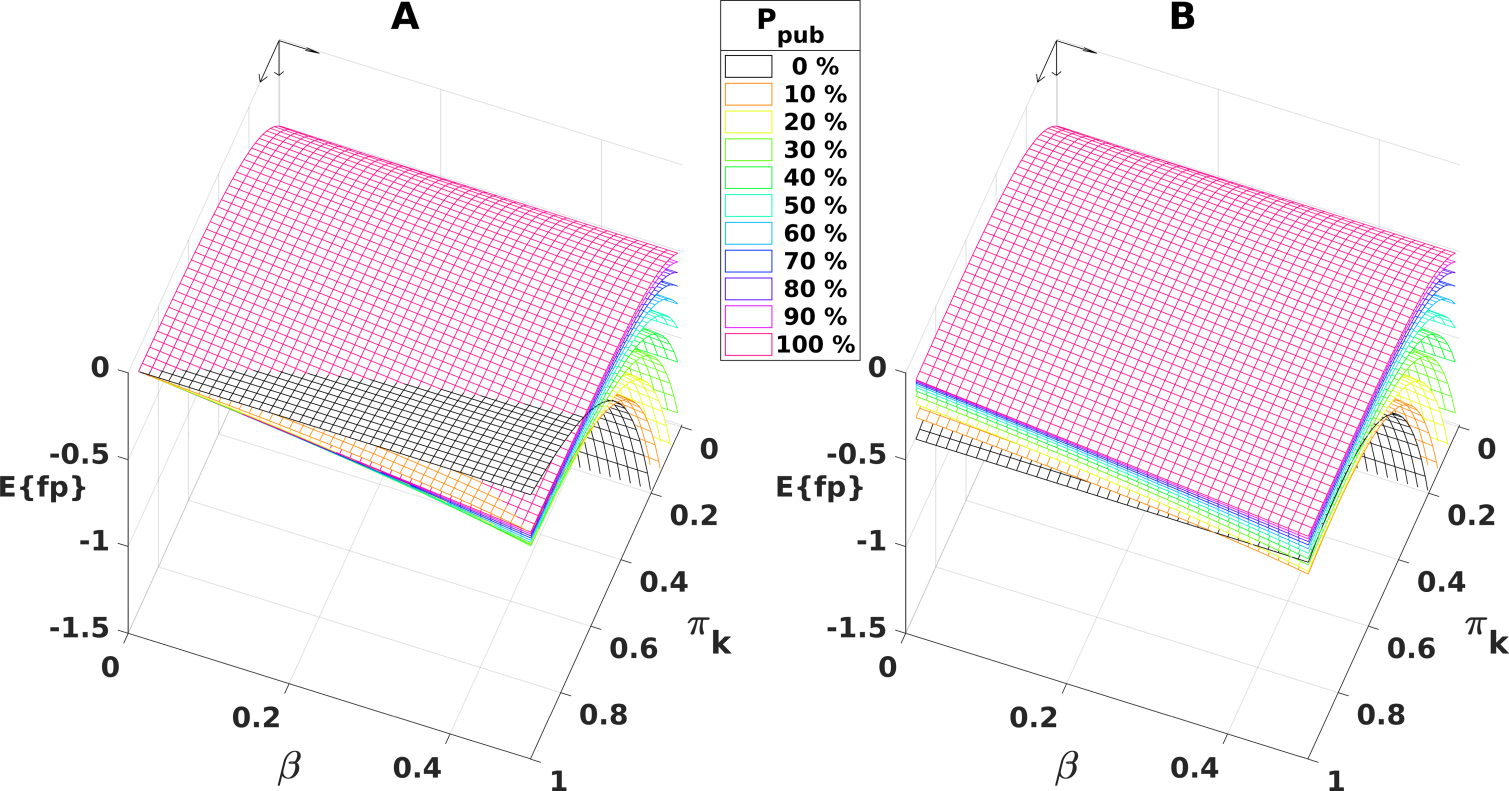
Expected number of false positive results E{fp} as a function of pre-study information, π_k_ and β, for 11 publication probabilities. Fixed parameters: **n**_**F**_ = 10, **n**_**t**_ = 10, **α** = 0.05, **δ** = 2. For a better presentation, the coordinate origin is in the upper rear corner. **Panel A**: The uppermost magenta grid points indicate that, for the largest set of parameter combinations (area: ~78%), the publication of all ‘negative’/null results leads to a favorable lower number of false positives (**E{fp}**). However, for parameter pairs with higher values of **π**_**k**_ and **β**, the uppermost black grid points (**P**_**pub**_ = 0%) indicate that any publication of ‘negative’/null results can be adverse regarding the number of false positives. However, studies represented by this area should be not conducted (see text). For publication probabilities between 0% and 100% (indicated by other colors), the statement remains the same. However, the area of ostensible beneficial effect of omitted publications is even smaller. **Panel B** shows the situation when minor errors in the testing order exist, that is, here the exchange of the first and second most probable factor. Additionally, this leads to significant increases of the expected number of false positives. There are no combinations of parameters left for which omitting publication is beneficial.

Fig 7 shows **E{fp}** as functions of **β** and **π**_**k**_ for 11 different publication probabilities, ranging from 0 to 1 (i.e., 0% to 100%). Note, the topmost area always represents the lowest expected number of false positives. In the figure, the magenta surface depicts the calculations for **P**_**pub**_ = 100%. This demonstrates that the publication of all results (**P**_**pub**_ = 100%) yields optimal results for most combinations of **β** and **π**_**k**_. Although there are also combinations of **β** and **π**_**k**_, for which it seems to be advantageous to not publish negative results (**P**_**pub**_ = 0%, black area in Fig 7A), we have to accept that these combinations represent heavily underpowered studies (i.e., studies with insufficient power for given pre-study information and knowledge). Therefore, these underpowered studies are not helpful to support the scientific gain. They should be simply avoided. However, there are clear indications that those studies are still published [8].

To examine the influence of utilizing pre-study knowledge, we simulated small errors in the testing order of the hypotheses (Fig 7B). Consistently, if pre-study information is not entirely exploited, it is always better to publish all results to avoid false positive results (all of the magenta surface area is topmost for all values of **β** and **π**_**k**_). The impact of **P**_**pub**_ on the total number of samples is similar to the impact on the expected number of false positive results.

## Discussion

We present a model of the scientific process as a whole to show that published results from individual scientific studies may have an impact on the direction of research. That is, false ‘negative’/null results can lead to unnecessary (follow-up) experiments. For modeling, it was assumed that a research problem is solved by testing several alternative hypotheses, consecutively. Our initial model describes the relationship between sample size, power, significance, and the expected number of false positives. It was subsequently extended to include any number of research teams, alternative hypotheses, and different probabilities to publish ‘negative’/null results. Therefore, the probability and effect of publishing false ‘negative’/null results were included. Additionally, several adjustments were added to reflect deviations from good scientific practice (GSP).

This extended model was used to answer two questions: 1) is it possible to reduce the overall consumption of resources (e.g. animal lives or patients) within the scientific process as a whole by increasing the sample size (e.g. number of laboratory animals) in individual experiments and 2) under which circumstances and to what degree is the publication of ‘negative’/null results beneficial? To the best of our knowledge, our presented model considers all relevant elements and parameters necessary to address these questions.

For the first question, the model calculations demonstrate that greater sample sizes for an individual experiment can lead to a reduction in the overall resource consumption. For animal experiments, this is equivalent to a reduction in the overall number of laboratory animals. If deviations from GSP are just moderate (see below), the statistical power that minimizes the resource consumption (**E{n**_**total**_**}**) is above 80% (i.e., **β** < 20%) for most of the tested scenarios (Figs 1 and 3).

With respect to the second question, the model shows that not publishing ‘negative’/null results has always detrimental impacts on the overall resource consumption and the produces excess of false positive results. Only scenarios characterized by a combination of high pre-study probability and low statistical power, in which the reliability of the results is already limited, are exempted. The conclusion derived from these results is, again, to publish always ‘negative’/null results. This is in line with the research results of Nissen et al. [29], but contradicts those of McElreath and Smaldino [23], who claim ‘publication bias is not always an obstacle, but may have positive impacts,’ based on their model calculations. In contrast to McElreath and Smaldino [23], our model considers that an unpublished study testing a scientific hypothesis could well be repeated by another research team and leads to an unnecessary waste of resources, eventually. This explains the apparent contradiction.

Interesting to note, de Winter & Happee [25] analyzed the probability of publication of ‘negative’/null results under two very extreme conditions. Their model describes the impact of ‘selective publication’ in the meta-analytical investigation of one effect, the size of which is estimated. ‘Selective publication’ is defined as no ‘negative’/null result is published, in our definition **P**_**pub**_ = 0. Or to ‘publish everything’, in our definition **P**_**pub**_ = 1. Quantitative estimation of the effect size is not included in our model, because this would lead to a complexity which is not helpful to answer our main questions at all. Thus, we do not explicitly model the meta-analytical process and a quantitative comparison was not performed to results of de Winter & Happee’s [25]. However, van Assen et al. [26] responded directly to the publication of de Winter & Happee [25], because they suggest, that publishing everything is more effective than only reporting significant outcomes selectively. The results of our analysis can be qualitatively compared to the results of these two studies in respect to the impact of ‘selective publishing’ on resource consumption. We measured the resource consumption by the total number of samples and not by the number of published studies as de Winter & Happee [25] did. Indeed, van Assen et al. [26] recognized already that de Winter & Happee [25] should have included the consumption of resources in their model. If so, they would have come to the conclusion that ‘publishing everything’ is efficient, which we join.

Next, we did not take into account that the uncritical use in the sample size calculation of exaggerated published effect sizes lead to underpowered studies. Using smaller sample size in combination with not publishing ‘negative’/null results leads in turn to even more exaggerated estimation of effect sizes. This consideration stresses all the more the necessity of the publication of ‘negative’/null results and appropriate sample size determination based on a conservative estimation of sample sizes.

With respect to the applicability of our results it must be taken into account, that the degree of flexibility in analysis and thereby the possibility to bias vary between different research fields and methodologies. If in a field the bias is too strong, higher sample sizes may not as beneficial as our results indicate for scenarios with small biases.

Therefore, the question arises if our results hold if scenarios with more than moderate deviations from our basic model assumption and from GSP are considered. Obviously, for extreme detrimental assumptions, such as a bias in favor of positive results close to 100% (fraud), the publication of ‘negative’/null results will not have any effect, nor is it advantageous to use higher sample sizes. If all hypotheses are tested in parallel, higher sample sizes in individual experiments also do not lead to the minimization of overall resource consumption. However, they do still result in higher PPV and the second conclusion (i.e., the beneficial effect of publishing ‘negative’/null results) still holds. If Joyner et al. are right and the current focus of whole domains of biomedical research is inappropriate [33] (i.e., the pre-study probability of the tested hypotheses is very low), hardly any measure can improve the reproducibility and efficiency of science, including higher sample sizes. However, the publication of ‘negative’/null results could still help avoid false positive results and unnecessary resource consumption.

An extensive sensitivity analysis, incorporating diverse deviations, demonstrated that our two main conclusions remain valid for most scenarios (for a complete list of examined scenarios, see supporting information (S2 Text)). The scenario analyses reveal the order of experiments reflecting decreasing pre-study probabilities is of utmost importance for resource consumption. This stresses the relevance of using pre-study knowledge during scientific planning and questions the use of other criteria for research prioritization.

In our model we also study the consequences of a decreased significance level, i.e., **α** from 0.05 to 0.005. We demonstrate that a reduction of the statistical power is not recommended, because **β**_**min**_ will be lower in most of the investigated scenarios if **α** is decreased (S2 Text).

We are aware that our model does not reflect all possible research strategies encountered in practice. For example, the classical group sequential trial design that allows interim analysis [34] or the use of historical data in a Bayesian framework [35] may help reduce the resource consumption, while maintaining statistical error rates.

To examine additional interesting scenarios and adapt models for different research conditions and questions, the model scripts (including documentation) written in R (.R) and Matlab (.m) are provided (as described in SI 3).

## Conclusion

We identified at least two applications for our model. First, the results support a more stringent way to enforce adherence to good scientific practice (GSP). Scientific journal editors may enforce this by requesting a self-declaration from authors regarding adequate study design and disclosure of ‘negative’/null, or more generally, of all results independently of outcome. The second application aims at the improvement of incentive schemes for researchers so that the interests of individual researchers are congruent with the improvement of science. With this respect to its application our model can be combined with that of evolutionary or game theoretic models. Removing the barrier to publishing negative research findings would be an easy way to improve the science and award scientists the incentive of additional publications. Furthermore, our model could be combined with other models (e.g., evolutionary or based on game theory) that describe the actions of individual scientists, given a specific incentive scheme. Funding agencies, journals, and employers, such as universities and governmental bodies, are especially in demand to implement appropriate measures. These measures depend on the current state of science in different research areas.

Summarizing our results leads to the perception that our model has of course limitations. We assume that science is self-correcting in the long run. But there might be research fields where this is no true, because of a general lack of correction of results or missing rewards for replications in comparison to innovative work. Introducing replications is a necessary step to improve science in general. Therefore, models which make use of the economic and social science tools in order to investigate the influence of institutional changes on the behavior of scientist are needed, here. Those methods, as described by Gall et al [27], could yield important insight on how to improve the quality of those scientific fields.

Furthermore, an essential question in the biomedical field is how many replications of pre-clinical trials are indeed needed, to be convinced that translational studies (e.g., clinical trials) can be started. Our current model cannot answer this question, because only binary study outcomes are considered. Thus, a third study outcome has to be introduced into our model in the future: A study result could be regarded as inconclusive and, therefore, a replication is warranted. Then different criteria for the classification of study results as acceptance or rejection of a hypothesis or their inconclusiveness could be tested.

## Supporting information

S1 Text Equations. (PDF)

S2 Text Sensivity analysis. (PDF)

S3 Text Description R and matlab scripts. (PDF)

S1 File Rscripts using Equations of S1. (R)

S2 File R-scripts for the MC simulation. (R)

S3 File R-script calculates the expectation of the total number of samples, false positives, and of the scientific gain. (R)

S4 File R-script for plots for expectation of the total number of samples. (R)

S5 File CSV file containing the model parameter values. (CSV)

S6 File Matlab script for Fig 7. (M)
