# Supplementary material for: Simple changes of individual studies can improve the reproducibility of the biomedical scientific process as a whole
Source: PLoS One. 2018 Sep 12;13(9):e0202762. doi: 10.1371/journal.pone.0202762 (PMC6135363; doi:10.1371/journal.pone.0202762)
Supplement: S1 Text — (PDF) [file pone.0202762.s001.pdf]

Supporting Information 1 (S1 Text): Equations

Content

In this supplement, the derivations of formulas for the expectations of the scientific gain, the number of false positives, and the total number of samples needed in the entire research process are presented. For comprehensive explanation, the Monte Carlo simulation of the scenario presented in Fig 2 (main text) that was used to test the validity of the formulas for the expectations is described first. Subsequently, formulas for the above mentioned expectations are presented for the case that only one factor is tested (n_F =1). Then we advance to more comprehensive equations for any numbers of factors taken into consideration for a research problem. From these equations, general properties can be derived. Especially, it can be proven that the higher π_k, the higher the statistical power should be. The corresponding proof is located at the end of this supplement. In addition, formulas for more than one causal factor are presented. For better orientation, please be referred to the list of all relevant variables presented below.

List of model variables

$E\{.\}$	(statistical) expected value of (.)
F_i	potential factors to be tested
n_a	sample size in a single experiment
n_F	number of factors (hypotheses) considered for a research problem
n_{cF}	number of (unknown) causal factors (true hypotheses)
n_t	number of research teams
n_K	number of needed studies to finish research
n_{total}	total number of samples
n_p	number factors (hypotheses) tested in parallel
π_k	conditional pre-study probability (of F_i)
P_{pub}	probability to publish negative results
u	bias
α	significance level
β	false negative rate ($1-\beta$ = power)
β_{min}	minimal value of β regarding $E\{n_{total}\}$
δ	standardized effect size
ω_i	objective pre-study probability

Description of the Monte Carlo simulation

In our research scenario (main text, Fig 2) we assume that n_F factors are under consideration ($F_1, \dots, F_i, \dots, F_{n_F}$). One or more of these factors are causal for the research problem under investigation. These causal factor(s) are placed on rank(s) between 1 and n_F using information of hypothetical exploratory pilot studies. If F_1 is causal, then $F_1 = 1$, and if F_1 is non-causal, then $F_1 = 0$ and so on. The probability of $F_1 = 1$ is given by ω_1. In the Monte Carlo (MC)

simulation, this ranking is randomised in accordance with ω_i in each iteration. The resulting rankings are stored in a matrix, which dimensions are determined by the number of factors under consideration (n_F: columns) and the number of simulation runs (N: rows). If there is only one causal factor, the sum of the matrix elements of column i divided by N gives approximately ω_i.

$$\begin{vmatrix} F_1 = 1 & \dots & F_i = 0 & \dots & F_{nF} = 0 \\ \vdots & \ddots & \vdots & \ddots & \vdots \\ F_1 = 0 & \dots & F_i = 1 & \dots & F_{nF} = 0 \\ \vdots & \ddots & \vdots & \ddots & \vdots \\ F_1 = 0 & \dots & F_i = 0 & \dots & F_{nF} = 0 \end{vmatrix}$$

The simulation procedure starts with the first team (team 1) testing F_1:

If $F_1 = 1$, F_1 is tested positive with the probability $1 - \beta$. In this case and if the number of causal factors $n_{CF} = 1$, the number of true positives is increased by 1 and the research process stops. If F_1 is tested negatively (false) this result will be published with the probability P_{pub}. If published, all other teams know this negative result and F_2 is tested next. If the result is not published, team 2 will test F_1. The testing of F_1 is repeated until A) one team tests F_1 positively (we assume that all positive results are published), or B) one team publishes a negative result, or C) all teams have tested F_1 negatively. Every test conducted increases the number of total samples by the sample size needed for this single experiment.

If $F_1 = 0$, the factor is tested positively with the probability α. The number of false positives is then increased by 1. After falsification of the result (an intrinsic characteristic of our simplified scenario) all teams know that the positive result is false and F_2 is tested next. If F_1 is tested negatively, it will be published with the probability P_{pub}. If published, all other teams know the negative result and F_2 is tested next. If the result is not published, team 2 will test F_1. Again, testing of F_1 is repeated until A) one team tests F_1 false positively (we assume that all positive results are published), or B) one team publishes a true negative result, or C) all teams have tested F_1 negatively. Every test conducted increases the number of total samples by the sample size needed for this single experiment.

The process continues until either A) all factors are tested and all teams know the results (either by reading the publication or by testing themselves) or B) all truly causal factors (n_{CF}) were tested positively. During the simulation, all true and false positive results and all samples needed in the process are counted. We consider independence among the results obtained from different teams, i.e. false positive and false negative study outcomes are not correlated given the true status (causal/ not causal) of the factor.

It is, however, also possible to derive formulas for the three outputs of the Monte Carlo Simulation, i.e. expectations of the scientific gain, number of false positives, and total number samples. For this, we need to first introduce equations for the pre-study probabilities. These equations are governed by the parameter π_k, which represents the pre-study information.

Additionally, we introduced a different scenario. Here, more than one factor is tested in parallel by different research teams. The number of factors tested in parallel is denoted by n_p and usually $n_p < n_F$. If all n_p test results are published and the truly causal factor is not detected the next n_p

factors will be tested and so on until factors are tested of the truly causal factor is detected. If in contrast a negative test result is not published, one of the teams having all not tested the corresponding factor will repeat this test. As in scenario described above this testing is iterated until one test result is published or all team have tested the factor.

A further extension of the model allows for errors in the validation process. An error of the first kind in the validation means that a false positive result is canonized, i.e. the process stops with a false result. An error of the second kind in the validation means that the true factor cannot be found.

Derivation of equations

- **Pre-study probabilities**

We picture that n_t research teams consider n_F factors as potential solutions to a research problem, i.e. as causal for an effect under investigation. For simplification, we initially assume that there is only one causal factor ($n_{cF} = 1$) that explains the observed effect. An exploratory study is performed once which, together with an evaluation procedure, ranks the n_F factors according to their likelihood to be causal. Since the outcomes of the exploratory studies can be regarded as random variables, there is an objective pre-study probability that the causal factor is placed on rank i (ω_i) in this simulation study.

For interpretation of the model outcomes, three properties of ω_i are advantageous:

(i) the probabilities should be decreasing ($\omega_1 \geq \omega_2 \geq \cdots \geq \omega_{n_F}$) i.e. the ranking is optimal,

(ii) the conditional probability (π_k) for $F_i = 1$ given $F_1 = 0, \ldots, F_{i-1} = 0$ should be constant for all i, and

(iii) the asymptotic sum over all ω_i should be $\sum_{i=1}^{\infty} \omega_i = 1$.

It can easily be derived that:

(1) $\omega_i = \pi_k (1 - \pi_k)^{i-1}, i = 1, .., n_F, (0 \leq \pi_k \leq 1)$

fulfils (i) to (iii). Equation (1) fulfils also (ii) as the conditional probability is given by $\frac{\omega_i}{1 - \sum_{j=1}^{i-1} \omega_j}$.

- **Expectation the total number of samples ($E\{n_{total}\}$; $n_F = 1$)**

The only factor considered in this case (F_1) can be either causal or not. The expected number of studies performed on this factor is the weighted sum of the expected numbers for both cases. Let us first assume that F_1 is causal. The factor will be tested exactly i times, if the following conditions are fulfilled:

- F_1 was tested negatively $i - 1$ times

- none of the corresponding studies were published
- F_1 is tested positively with the i-th trial or tested negatively with that trial and the study is published.

The maximum number of studies is given by the number of research teams in the field (n_t). If F_1 is already tested by $n_t - 1$ teams and none of the test was published, then F_1 will be tested n_t times regardless of the outcome of the experiment.

The expectation of the number of studies for $F_i = 1$ is given by the following series:

(2) $n_{e,1} = \{(1 - P_{pub})\beta\}^{n_t - 1} n_t + \sum_{i=1}^{n_t - 1} i\{(1 - P_{pub})\beta\}^{i-1}(1 - \beta + P_{pub}\beta)$

This equation can be simplified using the series $\sum_i^{n_F} i\, {\pi_k}^i = \frac{n_F \pi_k{}^{n_F+2} + \pi_k - (n_F+1)\pi_k{}^{n_F+1}}{(1-\pi_k)^2}$.
We thus obtain:

(3) $n_{e,1} = \frac{1 - \{(1-P_{pub})\beta\}^{n_t}}{1-(1-P_{pub})\beta}$

Analogously we derive the expected number of studies $n_{f,1}$ for the case that $F_1 = 0$. Here, β is replaced by $1 - \alpha$, which is the probability to obtain a true negative result. This transformation yields:

(4) $n_{f,1} = \frac{1-\{(1-P_{pub})(1-\alpha)\}^{n_t}}{1-(1-P_{pub})(1-\alpha)}$.

The expected number of studies is then:

(5) $E\{n_K\}(\alpha, \beta, n_F, \pi_k, P_{pub}) = \omega_1 n_{e,1} + (1 - \omega_1) n_{f,1}$

The expectation of the total number of samples used in the research process is given by the product of the number of samples per study $n_a(\alpha, \beta, \delta)$ and the expectation of the number of studies.

(6) $E\{n_{total}\} = n_a(\alpha, \beta, \delta) \times E\{n_K\}(\alpha, \beta, n_F, \pi_k, P_{pub})$

- **Expectation of the number of false positives ($E\{fp\}; n_F = 1$)**

A false positive publication is of course only possible, if $F_1 = 0$, which is the case with the probability $1 - \omega_1$.

The probability that team i is testing F_1 positively is given by the probability α multiplied by the probabilities that the preceding $i - 1$ teams tested F_1 negatively $((1-\alpha)^{i-1})$ and did not publish these results $((1 - P_{pub})^{i-1})$: $\alpha((1-\alpha)(1-P_{pub}))^{i-1}$.

The probability that F_1 is tested positively by any one team is obtained by summing these probabilities over all n_t teams (in our model, it is not possible that F_1 is tested positive by more than one team).

Using the geometric series we thus obtain:

(7) $E\{fp\} = fp_1(1-\omega_1) = \{1-\{(1-P_{pub})(1-\alpha)\}^{n_t}\}\frac{1-(1-\alpha)}{1-(1-\alpha)(1-P_{pub})}(1-\omega_1).$

Here, $fp_1 = \{1-\{(1-P_{pub})(1-\alpha)\}^{n_t}\}\frac{1-(1-\alpha)}{1-(1-\alpha)(1-P_{pub})}$ is the expected number of false positives if $F_1 = 0$.

- **Expectation of the number of true positives, i.e. scientific gain ($E\{g\}$; $n_F = 1$)**

Similarly we derive the equation for the expected scientific gain, i.e. the number of true positives. Here $(1-\alpha)$ is replaced by β and $1-\omega_1$ by ω_1. Therefore

(8) $E\{g\} = \{1-\{(1-P_{Pub})\beta\}^{n_t}\}\frac{1-\beta}{1-(1-P_{pub})\beta}\omega_1 = g_1\omega_1$ holds.

Here, $g_1 = \{1-\{(1-P_{Pub})\beta\}^{n_t}\}\frac{1-\beta}{1-(1-P_{pub})\beta}$ is the expected number of true positives given $F_1 = 1$.

Introduction of bias into the equations

To introduce bias we rely on the approach of [1] as the "proportion of probed analyses that would not have been "research findings," but nevertheless end up presented and reported as such, because of bias" (denoted by u)[1]. In equations (3), (4), (7)and (8) β is therefore replaced by $\beta(1-u)$ and $1-\alpha$ by $(1-\alpha)(1-u)$. Thereby we obtain the following equations:

(9) $n_{e,1} = \frac{1-\{(1-P_{pub})(1-u)\beta\}^{n_t}}{1-(1-P_{pub})(1-u)\beta},$

(10) $n_{f,1} = \frac{1-\{(1-P_{pub})(1-\alpha)(1-u)\}^{n_t}}{1-(1-P_{pub})(1-\alpha)(1-u)},$

(11) $fp_1 = \{1-\{(1-P_{pub})(1-\alpha)(1-u)\}^{n_t}\}\frac{1-(1-\alpha)(1-u)}{1-(1-\alpha)(1-u)(1-P_{pub})},$

(12) $g_1 = \{1-\{(1-P_{pub})\beta(1-u)\}^{n_t}\}\frac{1-\beta(1-u)}{1-(1-P_{pub})\beta(1-u)}.$

In order to determine the impact of β and α on scientific gain and false positive rate, respectively we differentiate (11) and (12).

The derivation of (11) with respect to α yields:

(13) $\frac{\partial fp_1}{\partial\alpha} = n_t\{(1-P_{pub})(1-u)\}^{n_t}(1-\alpha)^{n_t-1}\frac{1-(1-\alpha)(1-u)}{1-(1-\alpha)(1-u)(1-P_{pub})} + \{1-\{(1-P_{pub})(1-\alpha)(1-u)\}^{n_t}\}\frac{(1-u)P_{pub}}{(1-(1-P_{pub})(1-u)(1-\alpha))^2} \geq 0.$

The derivation of (12) with respect to β yields

(14) $\frac{\partial g_1}{\partial\beta} = -n_g(1-P_{pub})(1-u)^{n_t}\beta^{n_t-1}\frac{1-\beta(1-u)}{1-(1-P_{pub})\beta(1-u)} -\{1-\{(1-P_{pub})(\beta(1-u))\}^{n_t}\}\frac{(1-u)P_{pub}}{(1-(1-P_{pub})(1-u)\beta)^2} \leq 0.$

These equations prove that fp_1 increases with α while g_1 decreases with β.

Equations for arbitrary number of factors ($n_F \geq 1$)

The formula for the total number of samples for any number of considered factors n_F is derived by the following consideration:
If $F_i = 1$, which is the case with the probability ω_i, the mean number of tests of this factor is given by $n_{e,1}$. In this case, the preceding $i-1$ non-causal factors were each tested $n_{f,1}$-times in mean. In the case that F_i was not recognized as the causal factor, which happens with the probability $1-g_1$, additional $n_F - i$ non-causal factors will be tested $n_{f,1}$-times in mean. In the case that none of the considered factors is causal $n_F \times n_{f,1}$ studies will be performed in mean. Thus

(15) $E\{n_{total}\} = n_a(\alpha,\beta,\delta) \times \{\sum_{i=1}^{n_F}\omega_i\{n_{e,1} + (i-1)n_{f,1} + (1-g_1)(n_F-i)n_{f,1}\} + (1-\sum_{i=1}^{n_F}\omega_i)n_F n_{f,1}\} = n_a(\alpha,\beta,\delta) \times E\{n_K\}.$

In order to use the parametrization of the pre-study probabilities, we introduce some abbreviations:

$f_1(\pi_k, n_F) = \sum_i^{n_F}\omega_i = 1-(1-\pi_k)^{n_F}$, and
$f_2(\pi_k, n_F) = \sum_i^{n_F} i\,\omega_i = \frac{n_F(1-\pi_k)^{n_F+1}+1-(n_F+1)(1-\pi_k)^{n_F}}{\pi_k}$.
With this notation the expected number of studies in equation (15) can be written as:

(16) $E\{n_K\} = f_1(n_{e,1} - n_{f,1}) + g_1(f_2 - n_F f_1)n_{f,1} + n_F n_{f,1}.$

The equation for the expected number of false positives is derived similarly and we obtain:

(17) $E\{fp\} = \sum_{i=1}^{n_F}\omega_i\{(i-1)fp_1 + (1-g_1)(n_F-i)fp_1\} + (1-\sum_{i=1}^{n_F}\omega_i)n_F fp_1$
$= fp_1\{(f_2-f_1) + (1-g_1)(n_F f_1 - f_2)\} + (1-f_1)n_F fp_1.$

Equation (17) shows that the number of false positives increases with β, because g_1 decreases with β and g_1 is the only term in (17) which depends on β. This also means that the absolute number of false positive results decreases with increasing sample size n_a and vice versa.

The expectation of the scientific gain is given by the product of the probability that the causal factor is among the n_F factors considered ($\sum_i^{n_F} \omega_i$) and the probability that a causal factor will be tested positively by one of the n_t teams (g_1):

(18) $$E\{g\} = \sum_{i=1}^{n_F} \omega_i \, g_1 = f_1 g_1$$

Parallel testing I

So far, we assumed that the research teams performed the studies consecutively. Let us now consider a scenario in which each factor is tested by all teams in parallel, but in which results are published consecutively. Then $n_{e,1} = n_t$ and $n_{f,1} = n_t$, while g_1 and fp_1 remain unchanged. Therefore, $E\{fp\}$ and $E\{g\}$ are unchanged, too, and the following equation is obtained for the expected number of studies:

(19) $$E\{n_k\} = \sum_{i=1}^{n_F} \omega_i \{i \, n_t + (1 - g_1)(n_F - i) n_t\} + (1 - \sum_{i=1}^{n_F} \omega_i) n_F n_t$$

Since in this scenario $n_{e,1}$ and $n_{f,1}$ do no longer depend on P_{pub}, publishing negative result would not increase the efficiency of research, i.e. decrease $E\{n_{total}\}$.

Parallel testing II (factors are tested in parallel, $P_{pub} = 1$)

Another possibility is that n_p factors are tested in parallel. Then the expectation for the number of studies is:

(20) $$E\{n_k\} = \sum_{i=1}^{n_F} \omega_i \{n_F + g_1 (\min(n_F, \mathrm{ceil}(\frac{i}{n_p})) - n_F)\} + (1 - \sum_{i=1}^{n_F} \omega_i) n_F.$$

The corresponding expectation for number of false positives is

(21) $$E\{fp\} = \sum_{i=1}^{n_F} \omega_i \, fp_1 \{\min(n_F, \mathrm{ceil}(\frac{i}{n_p})) - 1 + (1 - g_1)(\min(n_F, \mathrm{ceil}(\frac{i}{n_p})) - n_F)\} + (1 - \sum_{i=1}^{n_F} \omega_i) n_F fp_1$$

Equations for arbitrary number of causal factors ($n_{cF} \geq 1$)

The model can be extended to the case of more than one causal factor. In this case the research problem is solved, if all n_{cF} causal factors are detected.

In order to derive an equation for $E\{n_K\}$ for arbitrary n_{cF}, we need an expression for the probability that $F_i = 1$, if there were already $n_{cF} - 1$ causal factors among the $i - 1$ factors so far considered. Let us denote this probability by $\omega_i^{end, n_{cF}}$.

Furthermore, a formula is needed for the probability $P(j; n_F, n_{cF})$, that exactly j out of n_{cF} causal factors were among the n_F factors.

To obtain a formula for $\omega_i^{end,n_{cF}}$, let us consider an ordering of n_F factors such that the last of $n_{cF}-1$ causal factors is placed on position j, i.e. there are $n_{cF}-2$ causal factors on positions preceding j. The probability for such an ordering is $\omega_i^{end,n_{cF}-1}$. The probability that an additional causal factor is placed on position $i > j$ is given by $\pi_k(1-\pi_k)^{i-(n_{cF}-1)-1}$. We therefore obtain the following recursion formula:

(22) $\omega_i^{end,n_{cF}} = \omega_i^{end,n_{cF}-1}(1-(1-\pi_k)^{i-(n_{cF}-1)}) + \sum_{j=n_{cF}-1}^{i-1} \omega_j^{end,n_{cF}-1} \pi_k(1-\pi_k)^{i-n_{cF}},$

$n_{cF} > 1, i = n_{cF}, .., n_F$

The first summand of (22) takes the case into account that the last of $n_{cF}-1$ factors are placed on position i. The term $1-(1-\pi_k)^{i-(n_{cF}-1)}$ describes then the probability that the additional factor is placed on a position from 1 to $i-1$. The recursion base of 0 is given by $\omega_i^{end,1} = \omega_i = \pi_k(1-\pi_k)^{i-1}$.

To obtain a formula for $P(j; n_F, n_{cF})$, we consider that there are two ways to choose j out of n_{cF} causal factors among the n_F factors.

Either there were j out of $n_{cF}-1$ causal factors chosen among n_F factors and no additional causal factor will be chosen among the remaining $n_F - j$ factors or $j-1$ out $n_{cF}-1$ causal factors will be selected and one additional causal factor will be chosen among the $n_F-(j-1)$ remaining factors.

Therefore:

(23) $P(j; n_F, n_{cF}) = P(j; n_F, n_{cF}-1)(1-\pi_k)^{n_F-j} + P(j-1; n_F, n_{cF}-1)(1-(1-\pi_k)^{n_F-(j-1)}),$

for $j = 1, \ldots, (n_{cF}-1)$.This recursion formula starts with $P(j=0; n_F, n_{cF}) = ((1-\pi_k)^{n_F})^{n_{cF}}$ and $P(j=0; n_F, n_{cF}=0) = 1$, with $P(j; n_F, n_{cF}) = 0$ for $j > n_{cF}$ and $j < 0$.

The equation for $E\{n_K\}$ is then derived from the following consideration: If F_i is the last causal factor, exactly $n_{cF}n_{t,1} + (i-n_{cF})n_{f,1}$ studies will be performed in mean before the problem is solved, i.e. all n_{cF} factors are discovered. The research problem is not solved with the probability $1-g_1^{n_{cF}}$. Then additional $(n_F - i)n_{f,1}$ studies will be performed in mean. If there were only $j < n_{cF}$ causal factors among the n_F considered factors, the expectation of the number studies is equal to $(n-j)n_{f,1} + jn_{t,1}$. Therefore we obtain:

(24) $$E\{n_K\} = \sum_{i=n_{cF}}^{n_F} \omega_i^{end,n_{cF}} \left\{n_{cF}n_{e,1} + (i-n_{cF})n_{f,1} + \left(1-g_1^{n_{cF}}\right)(n_F - i)n_{f,1}\right\} + \sum_{j=0}^{n_{cF}-1} P(j; n_F, n_{cF})((n_F-j)n_{f,1} + jn_{e,1})$$

Similarly, it can be shown, that for the number of false positives and the scientific gain

(25) $E\{fp\} = \sum_{i=n_{cF}}^{n_F} \omega_i^{end}\{(i - n_{cF})fp_1 + (1 - g_1^{n_{cF}})(n_F - i)fp_1\} + \sum_{j=0}^{n_{cF}-1} P\,(j; n_F, n_{cF})(n_F - j)fp_1$

and

(26) $E\{g\} = \sum_{j=1}^{n_{cF}} P\,(j; n_F, n_{cF}) \sum_{k=1}^{j} k \binom{j}{k} g_1^k (1 - g_1)^{j-k}$,

hold, respectively.

Proof that the probability of a beta error (β_{min}) minimizing $E\{n_{total}\}$ decreases with $\pi_k$and n_F

We prove that the higher the pre-study probability (π_k), the higher the recommended statistical power $1 - \beta$ and therefore the sample size per study should be. The proof is given here only for $n_{cF} = 1$. However, as we will see, the proposition depends only on three widely applicable conditions. We further prove that the higher the number of considered factors n_F, the higher the recommended statistical power $1 - \beta$ and therefore the sample size per study should be. This proof could only is only given for $P_{pub} = 1$, $n_{cF} = 1$, n_p=1.

Definition of β_{min}:
The smallest probability of an error of the second kind β_m, which fulfills $E\{n_{total}\}(\beta_m; \pi_k, n_F, \alpha, \delta, P_{pub}) \le E\{n_{total}\}(\beta; \pi_k, n_F, \alpha, \delta, P_{pub})$ for all $\beta \in [0,1 - \alpha]$ for fixed $\pi_k, n_F, \alpha, \delta, P_{pub}$ is termed β_{min}. If $n_F, \alpha, \delta, P_{pub}$ are kept constant, β_{min} is a function of π_k denoted by $\beta_{min}(\pi_k)$. Similarly the expectation of the number of studies is denoted by $E\{n_k\}(\beta, \pi_k)$.

Proposition:
a) From $\pi_{k_1} < \pi_{k_2}$ follows $\beta_{min}(\pi_{k_2}) \le \beta_{min}(\pi_{k_1})$.

b) From $n_{F1} < n_{F2}$ follows $\beta_{min}(n_{F2}) \le \beta_{min}(n_{F1})$.

S1 Fig 1: Schematic curves of expectation of total the number of samples per research problem versus ß for two different pre-study probabilities π_{k_1} and π_{k_2} ($\pi_{k_1} < \pi_{k_2}$). The slope of the curves increase with π_k. Therefore the second curve $E\ \{n_{total}$(ß;π_{k_2})} is rising at least as long as the first curve $E\ \{n_{total}\}$ (ß; π_{k_1}) is rising. Then the new minimum can only be found left of the old or at the same position, which is equivalent to the proposition.

Proof:
For two different pre-study probabilities π_{k_1} and π_{k_2} there are two different functions of

expectation of the total number of samples used for a research problem(illustrated in S1 Fig 1). The proposition is proven, if

(27) $En_{total}(\beta_2, \pi_{k_2}) - En_{total}(\beta_{min}(\pi_{k_1}), \pi_{k_2}) \geq 0$

holds for all $\beta_2 > \beta_{min}(\pi_{k_1})$.

We demonstrate that (27) implies the proposition by assuming that β_{test} with $\beta_{test} > \beta_{min}(\pi_{k_1})$ is $\beta_{min}(\pi_{k_2})$. Then inequality (27) requires that $En_{total}(\beta_{test}, \pi_{k_2}) \geq En_{total}(\beta_{min}(\pi_{k_1}), \pi_{k_2})$, while the definition of β_{min} requires that $En_{total}(\beta_{test}, \pi_{k_2}) \leq En_{total}(\beta_{min}(\pi_{k_1}), \pi_{k_2})$. Both inequalities are satisfied only if $En_{total}(\beta_{test}, \pi_{k_2}) = En_{total}(\beta_{min}(\pi_{k_1}), \pi_{k_2})$.

However, if this is true, then β_{test} is not the smallest β minimizing En_{total}, i.e. $\beta_{min}(\pi_{k_2})$.

For illustration see S1 Fig 1. The difference in (27) corresponds to the vertical difference of intersection point 4 and intersection point 3 in S1 Fig 1.

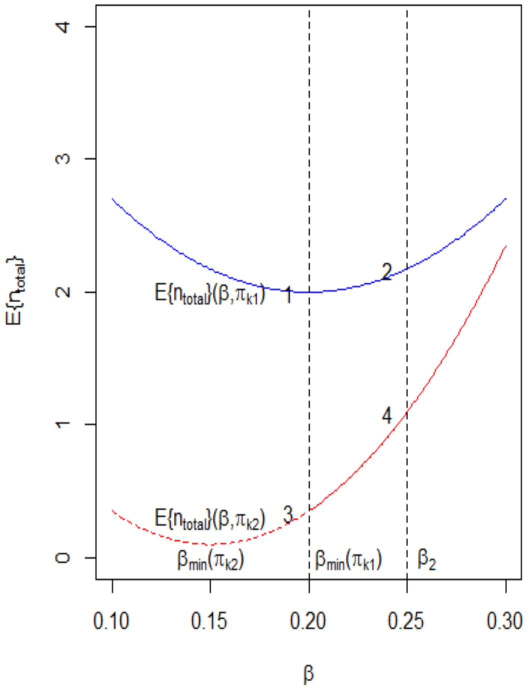

S1 Fig 1. Schematic curves of expectation of total the number of samples per research problem versus ß for two different pre-study probabilities π_{k_1} and π_{k_2} ($\pi_{k_1} < \pi_{k_2}$). The slope of the curves increase with π_k. Therefore the second curve $E\ \{n_{total}(ß;\pi_{k_2})\}$ is rising at least as long as the first curve $E\ \{n_{total}\}\ (ß;\ \pi_{k_1})$ is rising. Then the new minimum can only be found left of the old or at the same position, which is equivalent to the proposition.

To prove (27), we first demonstrate that three relations are sufficient to prove the proposition. We then show that these conditions apply to our model.

The relations are the following:

(i) $n_a(\beta;\alpha,\delta) \leq n_a(\beta_{min};\alpha,\delta)(\beta > \beta_{min})$,

(ii) $\frac{\partial E\{n_K\}}{\partial \pi_k} \leq 0$, and

(iii) $\frac{\partial^2 E\{n_K\}}{\partial \pi_k \partial \beta} \geq 0$

Relation (i) confirms that for higher power a bigger sample size is needed if everything else is kept constant. Additionally, it is assumed that n_a is constant throughout the research process. It implies additionally that prior probabilities are not directly used in the sample size determination. Relation (ii) means that we need less studies if our pre-study information is increased. Relation (iii) means that the impact of the statistical power on the number studies is higher, if the causal factors are tested earlier in the research process, which is more frequently the case the greater the pre-study information.

From (i) and the definition of β_{min} follows $E\{n_K\}(\beta_2,\pi_{k_1}) - E\{n_K\}(\beta_{min}(\pi_{k_1})) \geq 0$.
From (iii) follows:

(28) $$E\{n_K\}(\beta_2,\pi_{k_2}) - E\{n_K\}(\beta_{min},\pi_{k_2}) = \int_{\beta_{min}(\pi_{k_1})}^{\beta_2} \frac{\partial E\{n_K\}}{\partial \beta}(\beta,\pi_{k_2})d\beta$$
$$\geq E\{n_K\}(\beta_2,\pi_{k_1}) - E\{n_K\}(\beta_{min},\pi_{k_1}) = \int_{\beta_{min}(\pi_{k_1})}^{\beta_2} \frac{\partial E\{n_K\}}{\partial \beta}(\beta,\pi_{k_1})d\beta \geq 0$$

From inequality (28) follows:

(29) $$E\{n_K\}(\beta_2,\pi_{k_2}) - E\{n_K\}(\beta_2,\pi_{k_1}) \geq E\{n_K\}(\beta_{min}(\pi_{k_1}),\pi_{k_2}) - E\{n_K\}(\beta_{min}(\pi_{k_1}),\pi_{k_1}))$$

Because of relation (ii) both sides of the inequality (29) are negative. Therefore the following transformation holds:

(30) $$n_a(\beta_2) \times \left(E\{n_K\}(\beta_2,\pi_{k_2}) - E\{n_K\}(\beta_2,\pi_{k_1})\right) \geq n_a(\beta_{min}) \times \left(E\{n_K\}(\beta_{min}(\pi_{k_1}),\pi_{k_2}) - E\{n_K\}(\beta_{min}(\pi_{k_1}),\pi_{k_1})\right).$$

Further transformation yields:

(31) $$n_a(\beta_2) \times E\{n_K\}(\beta_2,\pi_{k_2}) - n_a(\beta_{min}(\pi_{k_1})) \times E\{n_K\}(\beta_{min}(\pi_{k_1}),\pi_{k_2}) \geq n_a(\beta_2) \times E\{n_K\}(\beta_2,\pi_{k_1}) - n_a(\beta_{min}(\pi_{k_1})) \times E\{n_K\}(\beta_{min}(\pi_{k_1}),\pi_{k_1})) \geq 0.$$

This inequality equivalent to (27) and proves thereby the proposition.

The four intersection points in S1 Fig 1 illustrate inequality (29). The left side of the inequality corresponds to the vertical difference of intersection points 4 and 3, the second difference corresponds to that of intersection point 2 and 1. It means that the increase in the total number of samples is even greater from $\beta_{min}(\pi_{k_1})$ to β_2, if π_k is increased.

We now show that the relations (i), (ii) and (iii) apply to our model:

Relation (i) can be assumed to be the case for all statistical tests. Relation (ii) follows from the differentiation with respect to π_k of (16). In equation (16), two terms depend on π_k:

$$\frac{\partial E\{n_K\}}{\partial \pi_k} = \frac{df_1}{d\pi_k}(n_{e,1} - n_{f,1}) + g_1(\frac{df_2}{d\pi_k} - n_F \frac{df_1}{d\pi_k}).$$

The first term is always negative, because for $\beta \leq (1-\alpha)$ the inequality $n_{e,1} \leq n_{f,1}$ holds and from the definition of f_1 follows $df_1/d\pi_k \geq 0$. For the second term we need to show that

$$\frac{df_2}{d\pi_k} - n_F \frac{df_1}{d\pi_k} = \frac{1}{{\pi_k}^2}\{(1-\pi_k)^{n_F-1}(1+(n_F-1)\pi_k) - 1\} \leq 0.$$

This is done by complete induction. The base clause yields $\frac{df_2}{d\pi_k} - n_F \frac{df_1}{d\pi_k} = 0$ for $n_F = 1$. The induction step yields

$$(\frac{df_2(n_F)}{d\pi_k} - n_F \frac{df_1(n_F)}{d\pi_k}) - (\frac{df_2(n_F+1)}{d\pi_k} - (n_F+1)\frac{df_1(n_F+1)}{d\pi_k}) = n_F(1-\pi_k)^{n_F-1}$$

and proves thereby $\frac{\partial EK}{\partial \pi_k} \leq 0$.

Relation (iii) is equivalent to

$$\frac{\partial^2 E\{n_K\}}{\partial \pi_k \partial \beta} = \frac{\partial}{\partial \pi_k}(f_1 \frac{\partial n_{t,1}}{\partial \beta} + \frac{\partial g_1}{\partial \beta}(f_2 - n_F f_1)) \geq 0.$$

This equation holds, because of $\frac{\partial n_{e,1}}{\partial \beta} \geq 0$ and $\frac{\partial g_1}{\partial \beta} \leq 0$.

Properties (ii) and (iii) for parallel testing

It can easily be seen that conditions (i-iii) apply also if parallel testing (I) of one factor by all teams is assumed. The proof can be derived if (19) is differentiated with respect to π_k and β.

A bit more complicated is the case of n_p factors tested in parallel (II). Here the proof is given for P_{pub}=1, only. Then the derivation of (20) yields:

$$\frac{\partial E\{n_k\}}{\partial \pi_k} = -g_1\{n_p^2 \sum_{j=1}^{floor(n_F/n_p)-1} j\,(1-\pi_k)^{n_p j-1} + n_p floor(n_F/n_p)(1-\pi_k)^{n_p floor(n_F/n_p)-1}(n_F - n_p floor(n_F/n_p))\}$$

Since all summands in the curly bracket are positive the derivation is negative and condition (ii) holds. Condition (iii) follows then directly from $\frac{\partial g_1}{\partial \beta} \leq 0$.

Proof of proposition b)

We start again with a sufficient condition for proposition b). Such a condition is given by

(32) $\mathrm{En}_{\mathrm{total}}(\beta_2, n_F + 1) - \mathrm{En}_{\mathrm{total}}(\beta_{\min}(n_F), n_F + 1) \geq 0$.

We show that (32) is fulfilled, if

(33) $\frac{\partial \ln E\{n_k\}(n_F)}{\partial \beta} \leq \frac{\partial \ln E\{n_k\}(n_F+1)}{\partial \beta}$ is true.

From (33) follows

$\ln(E\{n_k\}(n_F, \beta_2) - \ln(E\{n_k\}(n_F, \beta_{\min})) \leq \ln(E\{n_k\}(n_F + 1, \beta_2) - \ln(E\{n_k\}(n_F + 1, \beta_{\min}))$,

which implies:

$\ln(E\{n_k\}(n_F, \beta_2)/E\{n_k\}(n_F, \beta_{\min})) \leq \ln(E\{n_k\}(n_F + 1, \beta_2)/E\{n_k\}(n_F + 1, \beta_{\min}))$,

and

$E\{n_k\}(n_F, \beta_2)/E\{n_k\}(n_F, \beta_{\min}) \leq E\{n_k\}(n_F + 1, \beta_2)/E\{n_k\}(n_F + 1, \beta_{\min})$.

From the latter inequality and the definition of the minimum

$\frac{n_a(\beta_2)}{n_a(\beta_{\min})} \geq E\{n_k\}(n_F, \beta_{\min})/E\{n_k\}(n_F, \beta_2) \geq E\{n_k\}(n_F + 1, \beta_{\min})/E\{n_k\}(n_F + 1, \beta_2)$

is derived. This inequality implies (32) (given the definition of the minimum).

We now need to demonstrate that (33) applies to the model. Let us assume that $P_{\mathrm{pub}} = 1$. Then

$\frac{\partial E\{n_k\}(n_F)}{\partial \beta} = \sum_i^{n_F} \omega_i \, (n_F - i)$ holds.

Since $E\{n_k\}(n_F + 1) = \sum_i^{n_F} \omega_i \, g_1(i - (n_F + 1)) + n_F + 1 = E\{n_k\}(n_F) + 1 - \sum_i^{n_F} \omega_i \, g_1$,

we obtain

$\frac{\partial E\{n_k\}(n_F+1)}{\partial \beta} = \frac{\partial E\{n_k\}(n_F)}{\partial \beta} + \sum_i^{n_F} \omega_i$ and

(34) $\frac{1}{E\{n_k\}(n_F+1)} \frac{\partial E\{n_k\}(n_F+1)}{\partial \beta} - \frac{1}{E\{n_k\}(n_F)} \frac{\partial E\{n_k\}(n_F)}{\partial \beta}$

$$= \frac{\frac{\partial E\{n_k\}(n_F)}{\partial \beta} E\{n_k\}(n_F) + E\{n_k\}(n_F) \sum_i^{n_F} \omega_i - \frac{\partial E\{n_k\}(n_F)}{\partial \beta} E\{n_k\}(n_F) - \frac{\partial E\{n_k\}(n_F)}{\partial \beta} + \sum_i^{n_F} \omega_i g_1 \frac{\partial E\{n_k\}(n_F)}{\partial \beta}}{E\{n_k\}(n_F)(E\{n_k\}(n_F) + \sum_i^{n_F} \omega_i g_1)} =$$

$$\frac{\sum_{i=1}^{n_F} i\omega_i}{E\{n_k\}(n_F)(E\{n_k\}(n_F) + \sum_i^{n_F} \omega_i g_1)} \geq 0.$$

Obviously (34) implies that (33) applies to the model. Thus proposition b) is valid for the model given $P_{pub} = 1$.

Reference

1. Ioannidis JP (2005) Why most published research findings are false. PLoS Med. 2(8):e124.
