# Supplementary material for: Simple changes of individual studies can improve the reproducibility of the biomedical scientific process as a whole
Source: PLoS One. 2018 Sep 12;13(9):e0202762. doi: 10.1371/journal.pone.0202762 (PMC6135363; doi:10.1371/journal.pone.0202762)
Supplement: S2 Text — (PDF) [file pone.0202762.s002.pdf]

Supporting Information 2 (S2 Text): Sensitivity analysis

Content

In this supplement, some additional analysis regarding parameter sensitivity is presented. Used variables are explained in S1 Text.

Sensitivity analysis

In order to investigate how sensitive our results are when varying the parameters u, $P_{pub,}$ and n_p (number of hypotheses tested in parallel), we performed a sensitivity analysis. Additionally, we introduced the possibilities that a positively tested true hypothesis will be falsely rejected in the further research (validation, probability β_v) or a positively tested false hypothesis will be falsely accepted (probability α_v).

In this section the following parameters were fixed unless otherwise stated:

δ=2, α=0.05, P_{pub}=1, β_v= α_v=0, n_p=1,u =0, n_t=10 (initial parameter set). A comparison of different significance levels (α=0.05 vs α=0.005) is located at the and of this section

S2 Fig 1 shows that for large set of parameter values similar curve shapes result for the total number of samples as function of the probability of a beta-error. S2 Fig 2 shows the starting point of the sensitivity analysis: The area where optimization is possible given the initial parameter set. Here, optimizations A) finding a β minimizing the total number of samples and B) maximizing the efficiency $E\{g\}/E\{n_{total}\}$. S2 Figs 3-8 show that there are less combinations of parameter values (= domain of parameter space) that allow for an optimization as described above if the research community deviates from the good scientific practise. However, a large set of combination remains that allow for an optimization if the deviations are small or moderate.

S2 Fig 3 shows the area of the parameter domain in which β_{min}<0.2 for n_p=1,2,10. From this figure it can be derived that as long as the number of hypotheses tested in parallel (n_p) is considerably smaller than that of all hypotheses taken into consideration (n_F). For instance, for n_F=10 and n_p=1in all cases where π_k>0.31 there is β_{min}<0.2, if n_p=2 β_{min}<0.2 holds for π_k>0.39. If n_F=40 and n_p=1 leads to β_{min}<0.2 for all π_k>0.08, for n_p=2 β_{min}<0.2 for all π_k>0.08, and for n_p=10 β_{min}<0.2 for all π_k>0.14. Finally, if 100 hypotheses are considered (n_F=100), then for n_p=1 and π_k>0.03 β_{min} is smaller then 0.2, while for n_p=2 and n_p=10 the same is true for π_k>0.04.

S2 Fig 4 illustrates the influence of the parameter u, which represents the amount of bias in favor of positive results. It shows that the decreasing size of the area where β_{min}<0.2 when u increases u. However, even if u=0.5 the decrease is moderate. For ten considered hypotheses (n_F=10) in order to obtain β_{min}<0.2 the value of π_k must be greater than 0.53. For n_F=100 π_k>0.06 is required for that purpose instead of π_k>0.03 for u=0. In contrast for u=0.95 and n_F=100 π_k>0.44 is required.

S2 Fig 5 similarly shows the same for the parameter P_{pub}: while the nonpublication of negative studies results in more samples in general, the impact on the area where $\beta_{min}<0.2$ is only slightly decreased, as long at least 50 % of the negative results are published.

Another possible deviation from the good scientific practice is simply to repeat an experiment if there is a “negative” result. This resembles the questionable research practice described by Bakker et al. [1]. The impact of such a procedure is shown in S2 Fig 6.

S2 Fig 7 illustrates the effect of errors of the first kind in the validation leading to the canonization of false positives. The probability of such an error is denoted by α_v. The figure demonstrates that the impact of small probabilities α_v is small.

S2 Fig 8 illustrates the effect of errors of the second kind in the validation leading to the acceptance of false negatives. The probability of such an error is denoted by β_v. The figure demonstrates that the impact of small β_v is small.

S2 Fig 9 displays the effect of changing the significance level from 0.05 to 0.005. As expected a change from $\alpha=0.05$ to $\alpha=0.005$ results in higher $E\{n_{total}\}$ and in most of the cases the β minimizing $E\{n_{total}\}$ is lower.

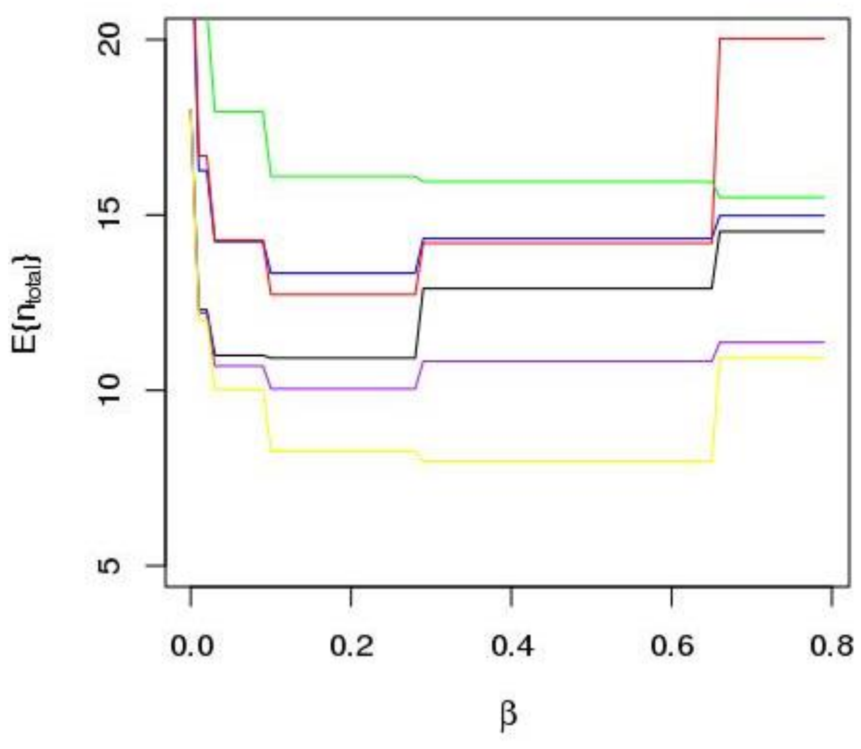

S2 Fig 1. Total number of samples as function of β (=1-power) ($n_F=10$). The black line represents the scenario with no parallel testing, full publication of negative results and no deviation from the good scientific practice. Some additional parameter changes are represented by the other lines:. Scenarios in which more than one scientific hypothesis are tested in parallel by different research teams ($n_p>1$) are considered. The scenario where two hypotheses are tested in parallel is represented by the blue line. The scenario where three hypotheses are tested in parallel is represented by the green line. The case of u=0.3 is represented by the purple line. Another

scenario of bias (the experiment is once repeated, if the first test result was negative) is displayed by the yellow line. Not publication of negative results in 20% of the cases is represented by the red line.

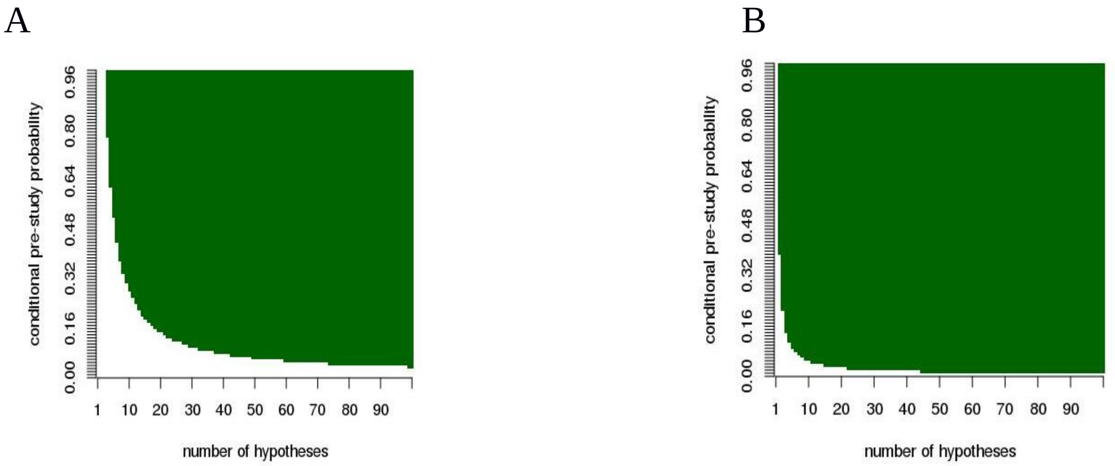

S2 Fig 2.

Panel A: Green areas indicate domains of the parameter space in which β_{min} – the probability of an beta-error globally minimizing E{n_{total}}- is lower than 0.2, i.e. in this area we find a useful minimum.

Panel B: Green areas indicate domains of the parameter space in which β_{max} – the probability of an beta-error globally maximizing E{g}/E{n_{total}}- is lower than 0.2.

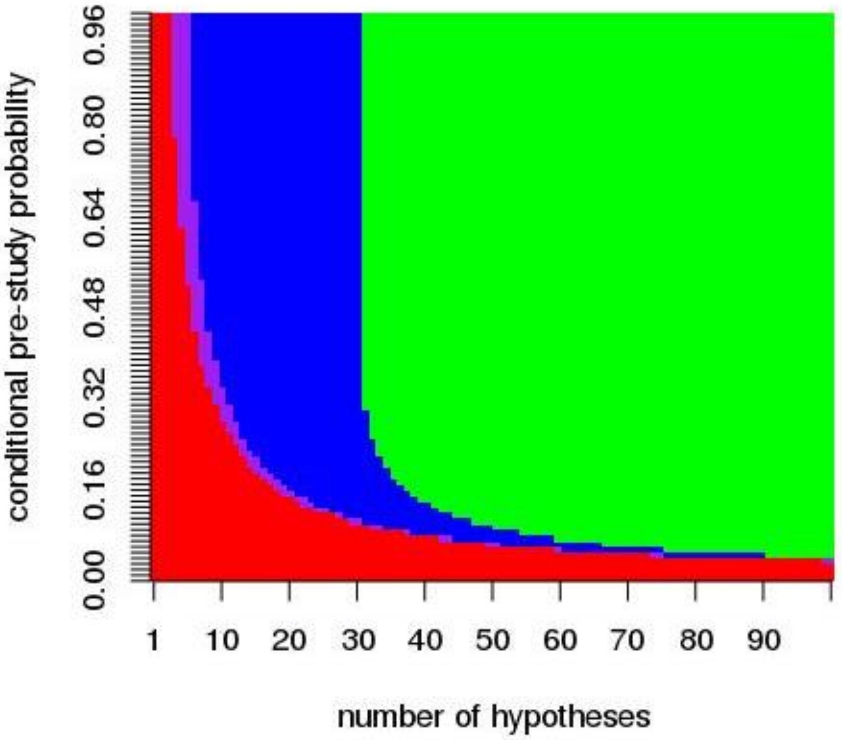

.

S2 Fig 3. The green area indicates the domain of the parameter space in which $\beta_{min}<0.2$, if n_p =10 (number of hypotheses tested in parallel) , the combined area colored green and blue indicate $\beta_{min}<0.2$ for n_p =2, the combination of green, blue, and purple areas (i.e. not the red area) indicate the same for n_p =1 (basic model assumption).

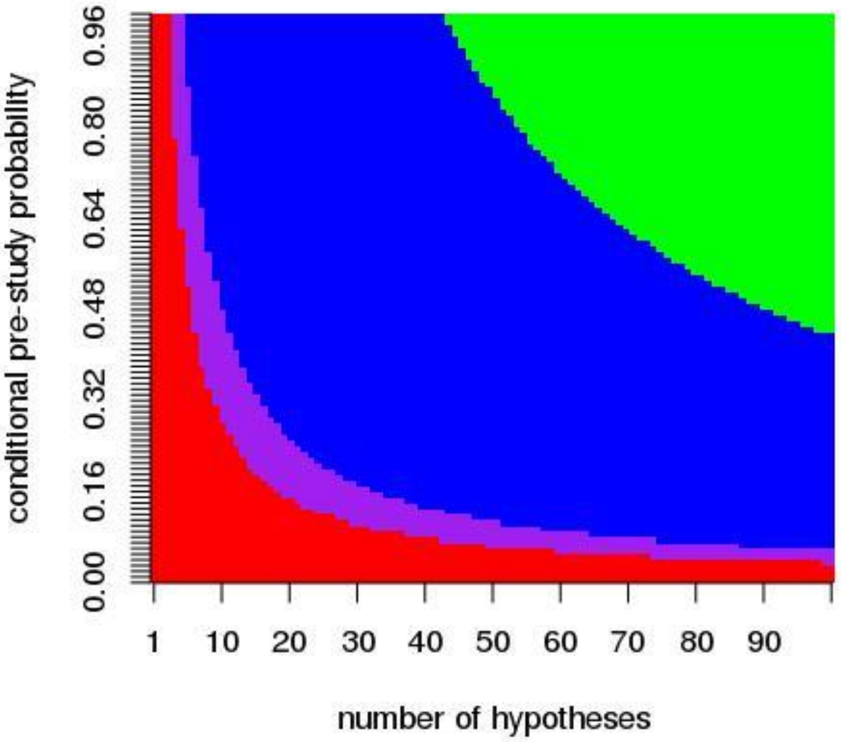

S2 Fig 4. The green area indicates the domain of the parameter space in which $\beta_{min}<0.2$, if u=0.95, green and blue areas indicate $\beta_{min}<0.2$ for u =0.5, green, blue, and purple for u=0 (basic model assumption).

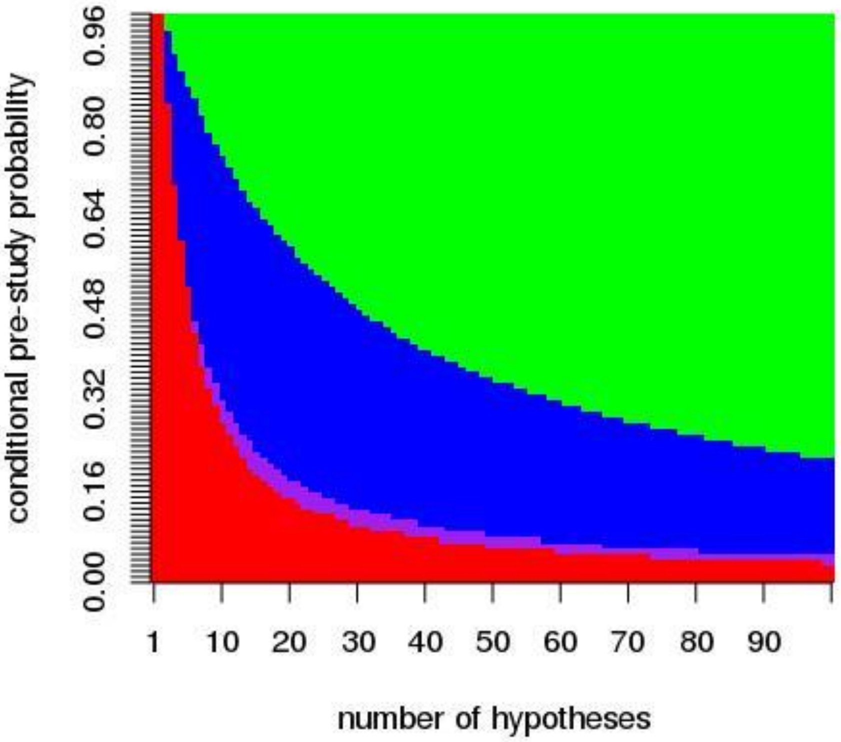

S2 Fig 5. The green area indicates the domain of the parameter space in which $\beta_{min}<0.2$, if $P_{pub}=0.05$, the combined area of green and blue indicate $\beta_{min}<0.2$ for P_{pub} =0.5, the combination of green, blue, and purple area indicate the same for P_{pub} =1, and, finally, the combinations of green, blue, dark blue, and purple areas for $P_{pub}=1$ (basic model assumption). Here are 10 teams assumed ($n_t=10$).

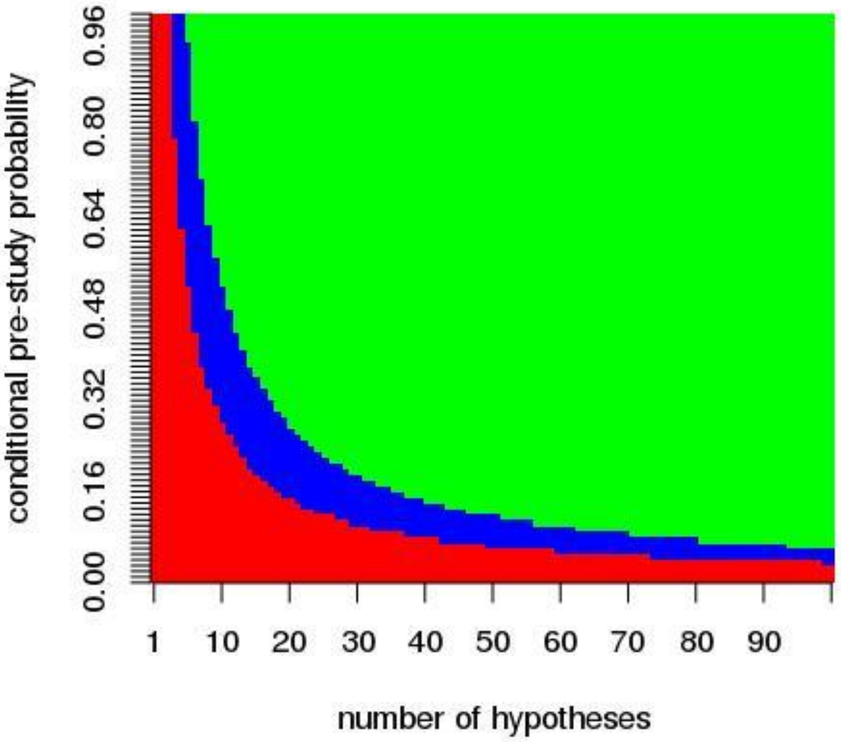

S2 Fig 6. The green area indicates the domain of the parameter space in which β_{min}<0.2 , if a negative test is repeated which is an extreme case of the questionable research practice in Bakker et al. [1] is applied, green and blue areas indicate β_{min}<0.2 if the basic assumptions are applied.

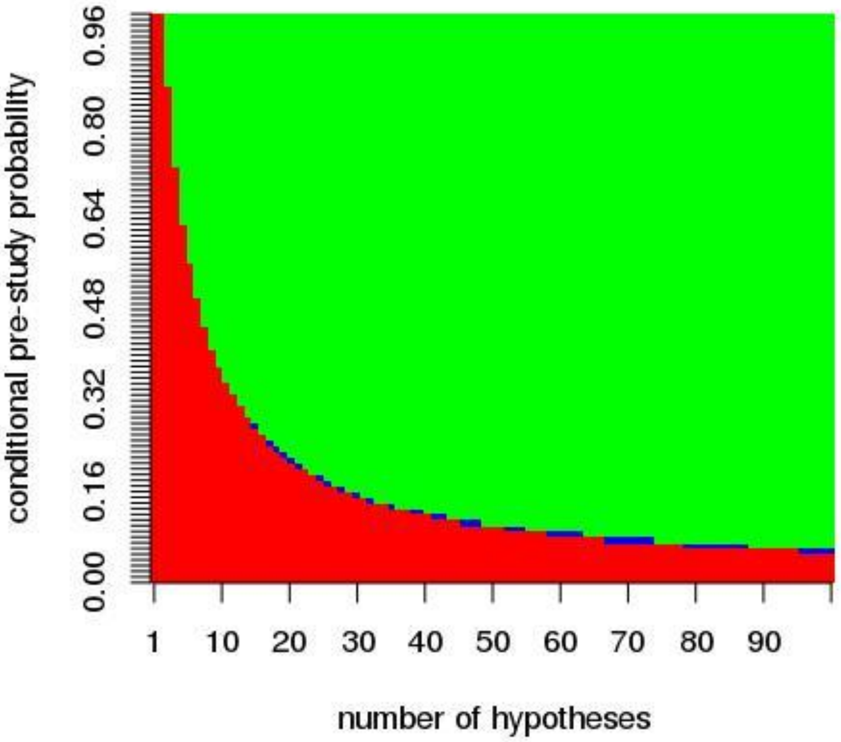

S2 Fig 7. The green area indicates the domain of the parameter space in which $\beta_{min}<0.2$, if $\alpha_v=0.05$, the combinations of green and blue areas indicate $\beta_{min}<0.2$ for $\alpha_v=0$.

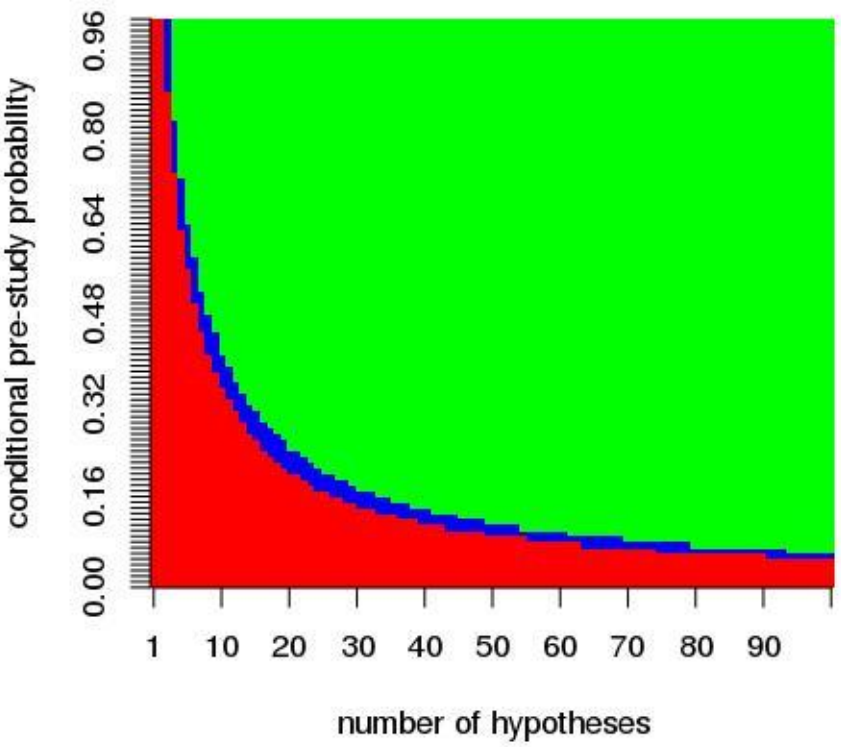

S2 Fig 8. The green area indicates the domain of the parameter space in which $\beta_{min}<0.2$, if $\beta_v=0.05$, the combination of green and blue areas indicate $\beta_{min}<0.2$ for, if $\beta_v=0$.

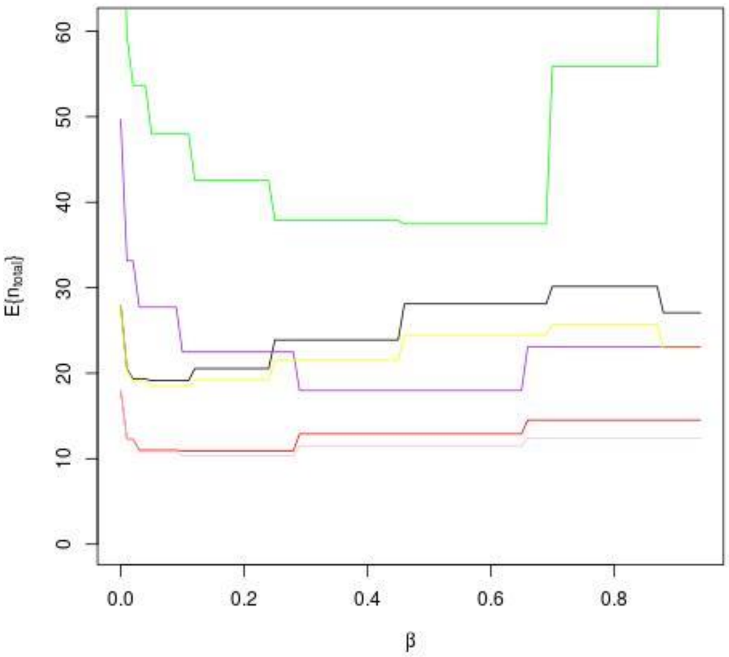

S2 Fig 9. Total number of samples as function of β (=1-power) (n_F=10) . Camparison of $\alpha = 0.05$ and $\alpha = 0.005$. Black line: $\alpha =$ 0.005, u=0 , P_{pub}=1. Red line: $\alpha =$ 0.05, u=0 , P_{pub}=1. Green line: $\alpha =$ 0.005, u=0 , P_{pub}=0. Purple line: $\alpha =$ 0.05, u=0 , P_{pub}=0. Pink line: $\alpha =$ 0.05, u=0.2 , P_{pub}=1. Yellow line : $\alpha =$ 0.005, u=0.2 , P_{pub}=1.

Reference

1. Bakker M, van Dijk A, & Wicherts JM (2012) The Rules of the Game Called Psychological Science. Perspect. Psychol. Sci. 7(6):543-554.
