# Supplementary material for: Simple changes of individual studies can improve the reproducibility of the biomedical scientific process as a whole
Source: PLoS One. 2018 Sep 12;13(9):e0202762. doi: 10.1371/journal.pone.0202762 (PMC6135363; doi:10.1371/journal.pone.0202762)
Supplement: S3 Text — (PDF) [file pone.0202762.s003.pdf]

**Supporting Information 3 (S3 Text):**

To examine additional interesting scenarios and adapt models for different research conditions and questions, the model scripts (including documentation) written in R (.R) and Matlab (.m) are provided:

- S4 File.R generates plots for expectation of the total number of samples, false positives, and efficiency dependent on beta.

- S5 File.csv: Parameters are imported by S4 File.R

- S3 File.R calculates the expectation of the total number of samples, false positives, and for the scientific gain. For this purpose it is required to upload S1 File.R and S2 File.R

- S6 File.m helps to examine scenarios with different values for p_pub and beta: This Matlab script calculates the expected number of false positives for all scenarios and displays these in two graphs: Panel A shows the results if all factors are tested in order of their pre-test probability and panel B shows the results if factor 1 and factor 2 are switched in order.
